# Trojan Horse Thiocyanate: Induction and Control of High Proton Conductivity in CPO-27/MOF-74 Metal–Organic Frameworks by Metal Selection and Solvent-Free Mechanochemical Dosing

**DOI:** 10.1021/acsami.1c06346

**Published:** 2021-06-17

**Authors:** Magdalena Lupa, Paweł Kozyra, Gabriela Jajko, Dariusz Matoga

**Affiliations:** Faculty of Chemistry, Jagiellonian University, Gronostajowa 2, 30-387 Kraków, Poland

**Keywords:** synthesis, mechanochemistry, metal−organic frameworks, proton transport, adsorption

## Abstract

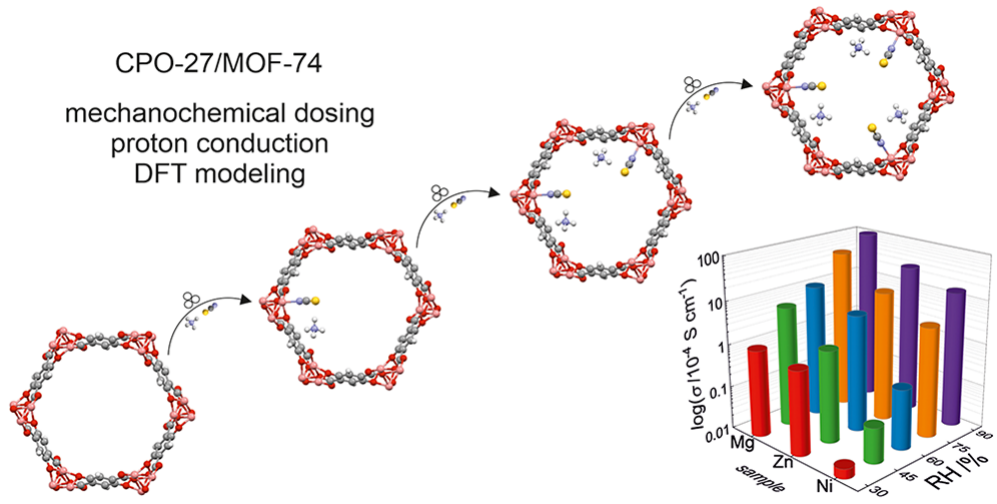

Proton-conducting metal–organic frameworks (MOFs) have been gaining attention for their role as solid-state electrolytes in various devices for energy conversion and storage. Here, we present a convenient strategy for inducing and tuning of superprotonic conductivity in MOFs with open metal sites via postsynthetic incorporation of charge carriers enabled by solvent-free mechanochemistry and anion coordination. This scalable approach is demonstrated using a series of **CPO-27/MOF-74** [M_2_(dobdc); M = Mg^2+^, Zn^2+^, Ni^2+^; dobdc = 2,5-dioxido-1,4-benzenedicarboxylate] materials loaded with various stoichiometric amounts of NH_4_SCN. The modified materials are not achievable by conventional immersion in solutions. Periodic density functional theory (DFT) calculations, supported by infrared (IR) spectroscopy and powder X-ray diffraction, provide structures of the modified MOFs including positions of inserted ions inside the [001] channels. Despite the same type and concentration of proton carriers, the MOFs can be arranged in the increasing order of conductivity (Ni < Zn < Mg), which strongly correlates with amounts of water vapor adsorbed. We conclude that the proton conductivity of **CPO-27** materials can be controlled over a few orders of magnitude by metal selection and mechanochemical dosing of ammonium thiocyanate. The dosing of a solid is shown for the first time as a useful, simple, and ecological method for the control of material conductivity.

## Introduction

Development of proton-conducting materials and gaining control of the conductivity are important both for fundamental understanding of charge transport phenomena as well as potential applications in sensors and polymer electrolyte membrane fuel cells (PEMFCs).^1,2^ Within the last decade, metal–organic frameworks (MOFs) emerged as a class of compounds suitable for these purposes, due to their high performance and designability.^3−6^ The literature contains several competing methods for inducing proton conductivity in MOFs which generally fall into two main categories. The first approach, known as the *de novo* synthesis, requires nonpolymeric building blocks and leads to the installation of Brønsted acidic centers either as linker pendant groups, guests, or terminal ligands. This approach, however, has serious limitations regarding inducing and control of conductivity in resultant frameworks. Direct introducing of linkers with acidic substituents is often hindered by their strong affinity toward metallic centers, which may consequently lead to undesired coordination and nonconducting products.^7^ Similarly, direct *de novo* introducing of charged guests or terminal ligands as proton carriers is often serendipitous as highly dependent on uncontrolled pH changes during framework assembly. A promising and effective alternative is offered by the second main approach toward proton-conducting MOFs that relies on postsynthetic modifications (PSMs) of preassembled frameworks.^8^ Within this approach, several proton-conducting MOFs were obtained via impregnation of parent MOFs with inorganic acids^9^ or protic organic molecules.^10^ Other distinct examples include organic linker modifications such as oxidation of thiol groups^11^ or ring-opening reactions with sultones.^12^ Finally, the combined *de novo* and PSM approaches can lead to a significant rise in conductivity, like in the MIL-101 platform in which pendant SO_3_H groups were first installed (MIL-101-SO_3_H)^13^ and then nonvolatile strong acid was introduced to form H_2_SO_4_@MIL-101-SO_3_H of excellent superprotonic conductivity.^14^ It is noteworthy, however, that all the abovementioned synthetic approaches toward proton-conducting MOFs usually involve complicated steps, require large amounts of solvents and energy, are difficult to upscale, and do not allow for control of proton conductivity.

As a remedy for typically complicated, costly, and nonecological syntheses in solution, a facile solvent-free mechanochemical approach, which is established in organic,^15^ organometallic^16^ and main group chemistry,^17^ and also enables large-scale synthesis,^18−23^ has increasingly become an alternative consideration for the preparation of various functional materials including porous MOFs.^24^ Even though mechanochemistry is not widely used to induce proton conductivity in MOFs, there are a few distinct reports in the literature. Among initial mechanochemical syntheses of proton-conducting MOFs were those described by Horike, Kitagawa, and others who carried out grinding of zinc oxide with azoles and orthophosphates, followed by the demonstration of intrinsic proton conduction of the resulting frameworks.^25,26^ Using the same mechanochemical approach for analogous systems, these researchers also reported the formation of a glassy state of a MOF that exhibited enhanced proton conductivity by 2 orders of magnitude as compared to its crystalline form.^27^ In 2015, we carried out a unique postsynthetic modification of {[Mn_2_(ina)_4_(H_2_O)_2_]·2EtOH}*_n_*^28^ (JUK-1) by grinding it with an ionic compound that yielded a proton-conducting {(NH_4_)_2_[Mn(ina)_2_(NCS)_2_]}*_n_*·*x*H_2_O (JUK-2).^29^ This fast and stoichiometric solid-state reaction led to a significant structural reconfiguration of the parent framework involving unzipping of crystalline JUK-1 bilayers to JUK-2 monolayers.^30^

While mechanochemical syntheses of MOFs are gaining impetus, the use of mechanochemical methods for tuning of either ionic or electronic conductivity of these materials has not been reported so far. In the literature, a remarkable tuning of electrical conductivity in MOFs was previously demonstrated for HKUST-1 infiltrated by a redox-active 7,7,8,8-tetracyanoquinodimethane (TCNQ) molecule.^31^ However, the tuning was achieved by controlling of exposure (immersion) time of the MOF in a saturated TCNQ/CH_2_Cl_2_ solution. It is worth noting that tunability of conductivity is not only necessary to obtain the value as high as possible (e.g., for PEMFCs), it is also important to tailor materials conductivity for some specific applications, e.g., in devices for energy conversion and information transfer, such as sensors, capacitors, memristors, transistors, and batteries.

In this work, we report a convenient and facile strategy for realizing postsynthetic tunable proton conductivity in MOFs by scalable solvent-free mechanochemistry. We demonstrate that grinding rigid **CPO-27/MOF-74(Mg, Zn, Ni)** materials with NH_4_SCN leads to a series of functionalized charged frameworks whose channels, filled with counterbalancing ions, become suitable for proton transport. Contrary to our precedent case study involving JUK-2,^29^ the mechanochemical reactions carried out in this work lead to virtually intact networks, and the highest proton conductivities of the modified frameworks 10-fold exceed that of JUK-2. The structures for the modified MOFs are provided by density functional theory (DFT) calculations and are corroborated by vibrational spectroscopy and powder X-ray diffraction (PXRD). The conducting properties of all materials are compared and discussed. Mechanochemical stoichiometric dosing of an ionic compound is shown for the first time as a useful, simple, and scalable method for the control of material conductivity.

## Results and Discussion

**Figure 1:**
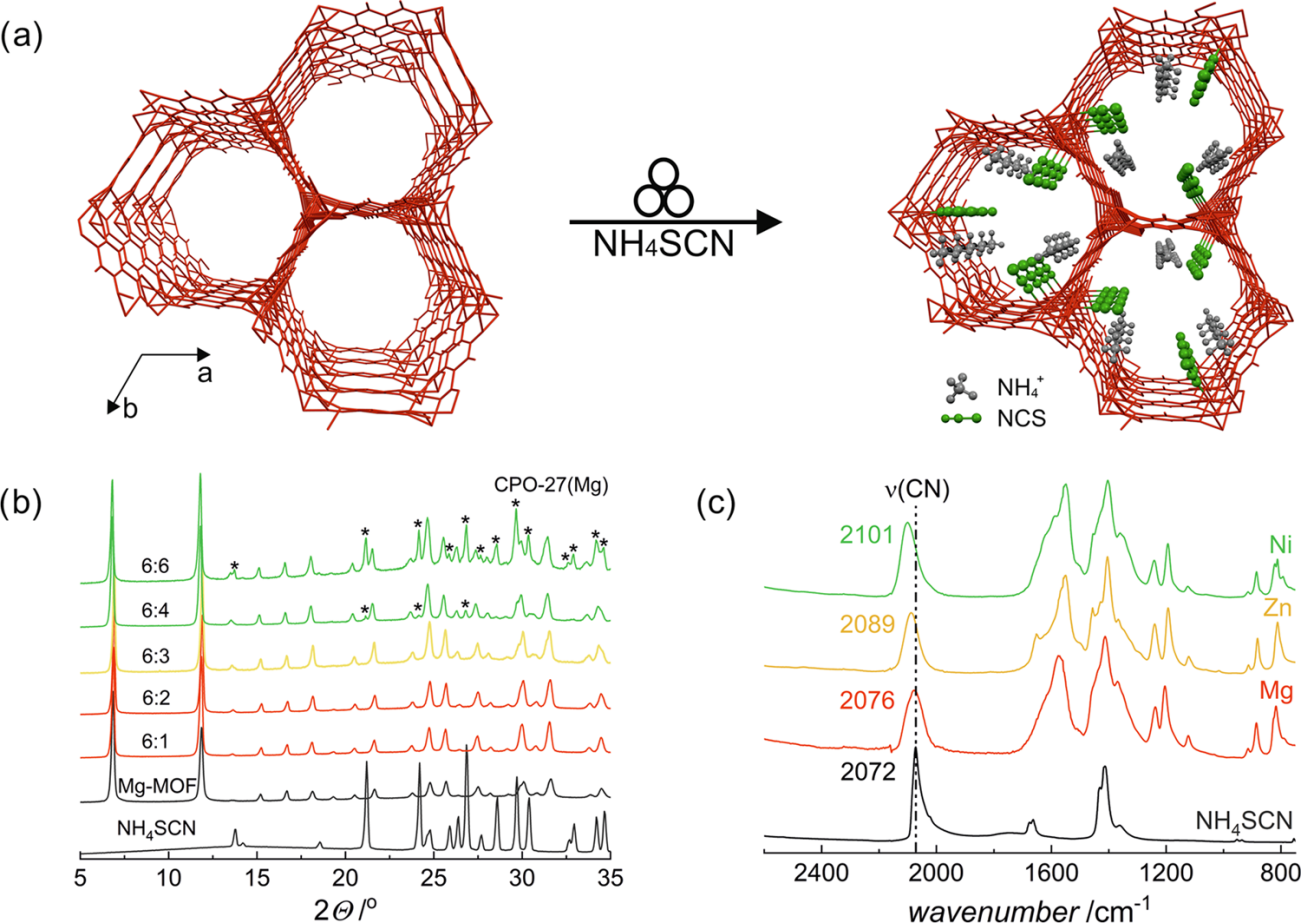
Solid-state reaction of **CPO-27** with NH_4_SCN: (a) general scheme for the mechanochemical synthesis of **CPO-27-NCS**; (b)  PXRD patterns of mixtures after grinding **CPO-27(Mg)** with various amounts of NH_4_SCN; 6:1, 6:2, 6:3, 6:4, and 6:6 (given as **CPO-27(Mg)** to NH_4_SCN stoichiometric ratio). Characteristic reflections of NH_4_SCN are labeled with “*”; and (c) FT-IR spectra of **CPO-27(Mg)-NCS**, **CPO-27(Zn)-NCS**, and **CPO-27(Ni)-NCS**. Numbers indicate wavenumbers (in cm^–1^) of the characteristic CN stretches.

The family of **CPO-27(M)** MOFs (M = Mn, Mg, Fe, Co, Ni, Cu, and Zn) is known for relatively stable structures containing one-dimensional channels with open metal sites.^32^*N*,*N*′-dimethylformamide (DMF) molecules coordinated to metallic centers in the as-synthesized materials can be replaced with methanol or water and removed by heating under vacuum.^33^ The channels can be decorated by functional molecules for the application of interest. Recent literature examples show that the coordinative insertion of *N*,*N*′-dimethylethylenediamine enhances CO_2_ adsorption,^34^ whereas the insertion of urea enables highly stable superprotonic conductivity.^35^ In this work, we have carried out new scalable modifications of **CPO-27** series and prepared three compounds with coordinated thiocyanates: (NH_4_)_3_[Mg_6_(dobdc)_3_(NCS)_3_(H_2_O)_3_]·*x*H_2_O (**CPO-27(Mg)-NCS**), (NH_4_)_3_[Zn_6_(dobdc)_3_(NCS)_3_(DMF)(H_2_O)_2_]·*x*H_2_O (**CPO-27(Zn)-NCS**), and (NH_4_)_3_[Ni_6_(dobdc)_3_(NCS)_3_(H_2_O)_3_]·*x*H_2_O denoted as **CPO-27(Ni)-NCS** (Figures 1 and S1). Regardless of the metal used, after grinding of **CPO-27** and NH_4_SCN with the addition of a small amount of EtOH, rigid frameworks remain virtually intact (as confirmed by PXRD patterns), whereas the evidence for the coordination of thiocyanate is provided by the infrared (IR) shift of the strong ν(CN) band from 2072 (for the initial NH_4_SCN) to 2076, 2089, and 2101 cm^–1^ for **CPO-27(Mg)-NCS**, **CPO-27(Zn)-NCS**, and **CPO-27(Ni)-NCS**, respectively (Figures 1, S1, and S2). The mechanochemical reactions are fast, stoichiometric, and occur with retainment of crystallinity. The maximal stoichiometry of these reactions was determined to be 6:3 (metal/thiocyanate ratio) for the three MOFs, as evidenced collectively by powder X-ray diffraction (XRD) and IR spectroscopy (Figures 1, S2, and S3). The maximally loaded **CPO-27-NCS** materials show practically no uptake of nitrogen and argon (Figures S4 and S5), as compared to high uptakes reported in the literature for the **CPO-27** materials, synthesized both in water and by solvent-free grinding, which indicates filling the pores of **CPO-27** by ammonium thiocyanate.^36,37^ The reactions carried out at higher stoichiometric ratios (6:4 and 6:6) gave mixtures of uncoordinated thiocyanate and **CPO-27-NCS** (observable by the appearance of extra X-ray reflections of NH_4_SCN and/or extra v(CN) IR bands), whereas the use of lower stoichiometric ratios (6:2 and 6:1) led to partial filling of channels with coordinated NCS^–^ and extra-framework NH_4_^+^ ions. The unique reactivity of the **CPO-27** materials opens possibilities for tuning of their properties by mechanochemical dosing of thiocyanates with other counterions.

To verify the possibilities of both formation of **CPO-27-NCS** and tuning of their proton conductivities in solution, **CPO-27(Mg, Zn, Ni)** MOFs were immersed in alcohol solutions (methanol or ethanol) containing NH_4_SCN at various SCN^–^ to MOF ratios (see the Supporting Information for details, Figures S6 and S7). Under no conditions we have tried the formation of **CPO-27-NCS** was observed, which proves the necessity of mechanical grinding in yielding **CPO-27-NCS**. Furthermore, given the recent demonstration of efficient gram-scale mechanosynthesis of **CPO-27(Zn)**,^36^ it is noteworthy that **CPO-27(Zn)-NCS** can be entirely prepared from simple nonpolymeric precursors in a two-step mechanosynthesis. The second mechanochemical step can be reversed by immersing the modified materials in alcohols for several days at elevated temperature, which leads to recovery of initial **CPO-27** materials upon removal of coordinated thiocyanates (Figure S8).

**Figure 2:**
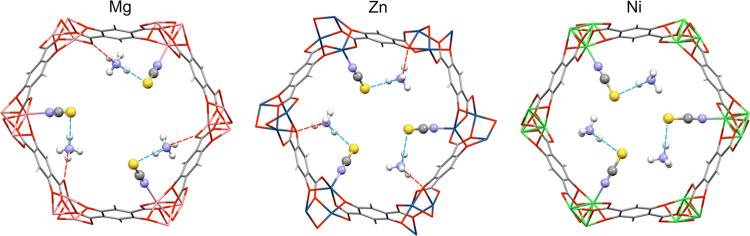
Structures of **CPO-27-NCS** optimized by periodic DFT+D methods, view along the [001] channels (Mg, Zn—full optimization; Ni—framework atoms frozen). Hydrogen bonds involving NH_4_^+^ ions are indicated by red (N–H···O) and cyan (N–H···S) dotted lines between donor and acceptor atoms. Color codes: C—gray, H—light gray, N—blue, O—red, and S—yellow.

Insights into the structures of the modified MOFs were provided by periodic DFT+D calculations (see the Supporting Information for details). Our experimental XRD data did not allow us to unambiguously locate the positions of NH_4_^+^ and SCN^–^ ions in the structure using Rietveld refinement (see the Supporting Information for details). We have taken under consideration the structures of **CPO-27(Mg, Zn, Ni)** with SCN^–^ anions inserted inside the [001] channels at the metal-to-thiocyanate ratio of 6:3. DFT optimizations were carried out for two possible linkage isomers with either S- or N-bound thiocyanate. In the simplified model, the **CPO-27** framework atoms were frozen. The calculated total energies for **CPO-27-NCS** and **CPO-27-SCN** optimized structures proved similar (for a given metal) and did not allow for the unambiguous discrimination between the two binding modes. Thus, full structural optimizations, which allowed a vibrational analysis, were performed for **CPO-27(Mg)-NCS**, **CPO-27(Mg)-SCN**, **CPO-27(Zn)-NCS**, and **CPO-27(Zn)-SCN**. Since the energies for the two linkage isomers within this approach were similar again, we have taken CN stretch wavenumbers as an indicator of the coordination mode of thiocyanate. The calculations clearly demonstrate that these wavenumbers are higher for M-NCS structures, with shorter CN bonds. To compare experimental IR wavenumbers for the modified **CPO-27** MOFs with those calculated for the two binding variants, we have scaled our method by performing vibrational analysis for the selected monomeric thiocyanate complexes containing either of the three Mg, Zn or Ni metals. The Cambridge Structural Database (CSD) database search gave a few structurally characterized compounds including two complexes of [Zn(NCS)_4_]^2–^ (CSD codes: MENBEM, MUDPIM, NESMOP)^38−40^ and [Ni(NCS)_6_]^4–^ (CSD codes: FILVAA, YIGGIH01)^41,42^ whose averaged experimental CN wavenumbers were 2085 and 2095 cm^–1^, respectively. Their comparison with the values calculated by our method (2113 and 2119 cm^–1^, respectively) allowed us to estimate the scaling factor for the method as 0.988 (experimental/calculated), which was applied for the calculated raw wavenumbers in the modified **CPO-27**, and the scaled values were compared with the experimental wavenumbers (see Table S1). The scaled CN stretches for Mg- and Zn-based structures with N-bound thiocyanates match the experimental values very well. Similarly, the CN stretches estimated for the modified **CPO-27(Ni)** indicate that the nickel-based structure contains the N-bound thiocyanate. All three **CPO-27-NCS** structures, optimized by DFT methods, are presented in Figure 2.

The optimized structures show significant differences in the location of ammonium ions inside the [001] channels (Figure 2). In all of the modified frameworks, ammonium ions are hydrogen-bonded with sulfur atoms of N-coordinated thiocyanates. These hydrogen bonds increase in strength as the average donor–acceptor distances (N···S) decrease in the order: Ni (3.35 Å) < Zn (3.27 Å) < Mg (3.14 Å), and the corresponding average N–H bonds are elongated: 1.066 Å (Ni) < 1.068 Å (Zn) < 1.089 Å (Mg) accordingly (Table 1). Apart from interactions with sulfur atoms, ammonium ions in **CPO-27(Zn, Mg)-NCS** are additionally stabilized by strong hydrogen bonds with intraframework oxygen atoms, which are also accountable for N–H bond elongations. The longer N–H bonds are, the higher proton mobility is inside MOF channels and, consequently, material conductivity.

**Table 1:** Hydrogen Bond Distances (Averaged, in Å) Involving NH_4_^+^ Ions Inside CPO-27-NCS

		Mg	Zn	Ni
N–H···S	*d*(NS)	3.14	3.27	3.35
	*d*(NH)	1.089	1.068	1.066
N–H···O	*d*(NO)	2.92	2.74	
	*d*(NH)	1.046	1.071	

**Figure 3:**
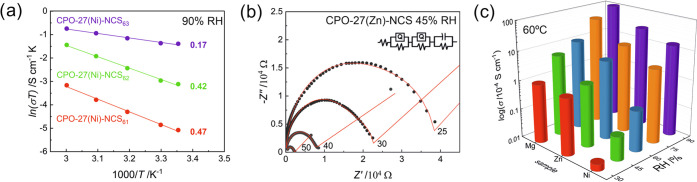
Proton conduction for **CPO-27-NCS**: (a) Arrhenius plots with activation energies indicated as numbers (in eV) after mechanochemical dosing of NH_4_SCN in **CPO-27(Ni)-NCS** (with 6:1, 6:2, and 6:3 metal/thiocyanate ratios); samples conditioned at 90% RH. (b) Temperature-dependent AC impedance plots for **CPO-27(Zn)-NCS** at 45% RH (maximal NH_4_SCN loading, numbers indicate temperatures in °C) with equivalent circuit used for fitting plots. (c) Proton conductivities at 60 °C and different RH values, all MOFs with maximal NH_4_SCN loading.

**Figure 4:**
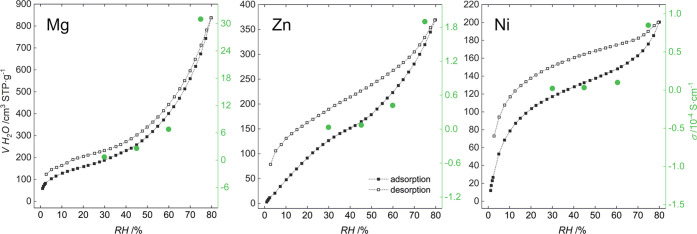
Proton conductivity versus water vapor adsorption–desorption isotherms for **CPO-27-NCS** (with maximal NH_4_SCN loading) at 25 °C.

In the literature, the recently reported studies for a single crystal of **CPO-27(Co)** showed that proton conduction of the material was anisotropic and occurred predominantly through one-dimensional channels (intrachannel proton conductivity at 25–30 °C was ∼10^–4^ S cm^–1^ in contrast to perpendicular directions with σ ∼ 10^–6^ S cm^–1^).^43,44^ It is justified to assume that the same intrachannel pathways are mainly responsible for proton conduction of other isoreticular **CPO-27** MOFs and their modifications with retained framework structures, such as the **CPO-27-NCS** materials in this work. During solvent-free grinding of **CPO-27(Co)** and ammonium thiocyanate, we have observed partial decomposition of **CPO-27(Co)** and the formation of [Co(NCS)_4_]^2–^ ions (the Vogel reaction),^45,46^ identifiable by the appearance of blue color (see Figure S11). However, the solvent-free reactions of **CPO-27(Mg, Zn, Ni)** MOFs with NH_4_SCN yielded **CPO-27-NCS** with ammonium proton carriers inside their unidirectional channels and induced high proton conductivity in the modified materials. This functionalization was enabled using thiocyanates as Trojan horse anions “carrying” counterbalancing protic cations. For proton conduction studies of the modified MOFs, alternating current (AC) impedance measurements were carried out using powdered samples pressed between metallic electrodes in a poly(tetrafluoroethylene) (PTFE) tube under different RH values from 30 to 90%, and variable temperatures from 25 to 60 °C (Figures 3 and S12–S16). The highest proton conductivities in the series, reaching 10^–2^ S cm^–1^ at high relative humidity (60 °C, 90% RH) and 10^–4^ S cm^–1^ already at 30% RH, were observed for **CPO-27(Mg)-NCS**. Despite the same type and concentration of proton carriers, the other two materials, **CPO-27(Zn, Ni)-NCS** showed lower values (Figure 3 and Table S2) and the whole series can be arranged in the decreasing order of conductivity: Mg > Zn > Ni. The series correlates well with the decreasing hydrophilicity of the MOFs, confirmed by their water vapor uptake isotherms (Figure 4). The isotherms also show the stability of the materials during adsorption–desorption cycling (Figure S24) as well as significant differences between the three MOFs including highest uptakes and conductivities for **CPO-27(Mg)-NCS** and the lowest for **CPO-27(Ni)-NCS**. The nanoconfinement of NH_4_SCN within the [001] channels of **CPO-27** materials has a double effect on conductivity: (i) it induces high proton conductivity of **CPO-27** through introducing extra-framework proton carriers and (ii) it modulates the ability of **CPO-27** to adsorb water molecules that are essential elements of conduction pathways. The latter strongly depends on the selection of a metal center in the **CPO-27** material. By metal selection (Mg, Zn, or Ni) and RH adjustment, proton conductivity of **CPO-27-NCS** can be controlled over 3 orders of magnitude (Figure 3c). It is likely that further finetuning might be achieved using various mixed-metal **CPO-27** frameworks, whose mechanochemical syntheses have been recently reported in the literature.^47^ Interestingly, apart from appropriate metal selection, proton conductivities of **CPO-27-NCS** can be additionally controlled by mechanochemical stoichiometric dosing of thiocyanate ions within the 6:1–6:3 metal-to-thiocyanate ratio range. Unattainable by immersion in solution, tunable proton conductivity over 1 order of magnitude was achieved for all **CPO-27-NCS** at 90% RH and 60 °C (Tables S3–S5 and Figures S19–S21). At lower temperatures, the tuning range depends on a metal center and is either slightly narrower or wider, with up to 2- (Zn), 6- (Mg), and 40-fold (Ni) increases of conductivity upon increase of the dose of NH_4_SCN from 6:1 to 6:3. The conductivities achieved for the largest 6:3 doses are among the highest reported for similar materials and significantly higher as compared to initial **CPO-27** MOFs (Table S6).

The linear regression of Arrhenius plots of the temperature-dependent proton conductivities of **CPO-27-NCS** provided activation energies (*E*_a_) for proton transport inside their microporous channels at various RH values (Figures 3, S17, and S22). For two materials in the series, that is **CPO-27(Mg, Ni)-NCS**, the activation energies did not exceed 0.4 eV in the whole RH range, which shows that proton conduction occurs by a Grotthuss-type hopping mechanism, involving water molecules and ammonium cations. By contrast, proton conduction of **CPO-27(Zn)-NCS** is characterized by the increased *E*_a_ values between 0.46 and 0.86 eV, which indicates the different mechanism of proton transport involving translational movements of NH_4_^+^ and H_3_O^+^ ions as well as their deprotonated counterparts (vehicle mechanism). In general, NH_4_^+^ conductors are promising materials for electrochemical devices such as fuel cells utilizing ammonia or ammonium salts.^48,49^ The different mechanism for **CPO-27(Zn)-NCS** is likely associated with partial coordination of zinc metal centers with DMF molecules, whose presence disrupts the hydrogen bond network inside channels for proton hopping.

## Conclusions

We have reported a convenient strategy for realizing tunable proton conductivity in metal–organic frameworks in which micropores are loaded with protic guest cations by solvent-free mechanochemistry. This scalable and facile approach is demonstrated using a series of **CPO-27/MOF-74** materials loaded with ammonium thiocyanate. Tunable proton conductivities over 4 orders of magnitude are achieved in the whole series, with values as high as 10^–2^ S cm^–1^. The strategy could be extended with other salts composed of protic ions and those capable of complexation. The targeted MOFs for such postsynthetic modifications should preferably contain undercoordinated open metal sites. The modified proton-conducting MOFs of good stability and high performance may have applications in devices such as fuel and solar cells as well as other devices for energy conversion and information transfer.

## Supporting Information Available

DFT optimized structure for **CPO-27(Mg)-NCS** (CIF)
DFT optimized structure for **CPO-27(Zn)-NCS** (CIF)
DFT optimized structure for **CPO-27(Ni)-NCS** (CIF)
Details of synthetic procedures, sample handling, physical measurements, and computational studies; additional IR, PXRD, EIS, and adsorption data (PDF)

## Supplementary Material

am1c06346_si_001.cif

am1c06346_si_002.cif

am1c06346_si_003.cif
