# Supplementary material for: Trojan Horse Thiocyanate: Induction and Control of High Proton Conductivity in CPO-27/MOF-74 Metal–Organic Frameworks by Metal Selection and Solvent-Free Mechanochemical Dosing
Source: ACS Appl Mater Interfaces. 2021 Jun 17;13(25):29820–6. doi: 10.1021/acsami.1c06346 (PMC8289229; doi:10.1021/acsami.1c06346)
Supplement: Supplementary file 4 — am1c06346_si_004.pdf [file am1c06346_si_004.pdf]

Supporting Information

Trojan horse thiocyanate: induction and control of high proton conductivity in CPO-27/MOF-74 metal-organic frameworks by metal selection and solvent-free mechanochemical dosing

Magdalena Lupa, Paweł Kozyra, Gabriela Jajko and Dariusz Matoga*

Faculty of Chemistry, Jagiellonian University, Gronostajowa 2, 30-387 Kraków, Poland
Corresponding author: dariusz.matoga@uj.edu.pl

Contents:

Synthetic procedures and control experiments 4

Details of physical measurements 6

Computational details 6

Rietveld refinement procedure 7

Figures and Tables 8

Figure S1. IR spectra (left): **CPO-27** as-synthesized (black) and **CPO-27(M)-NCS** (**CPO-27** after LAG with NH_4SCN at 6:3 molar ratio (red). PXRD patterns (right): **CPO-27** calculated from SC-XRD data (green), the as-synthesized bulk material (black), and **CPO-27(M)-NCS** (red). 8

Figure S2. IR spectra (left) and PXRD patterns (right) of mixtures after grinding **CPO-27** with various amounts of NH_4SCN; 6:1, 6:2, 6:3, 6:4, 6:6 (given as **CPO-27** to NH_4SCN ratio). Characteristic reflections of NH_4SCN are labeled with '*'. 9

Figure S3. IR spectra of mixtures after grinding **CPO-27** with various amounts of NH_4SCN; 6:1, 6:2, 6:3, 6:4, 6:6 (given as the **CPO-27** to NH_4SCN ratio) showing *v*(CN) range 10

Figure S4. N_2 adsorption measurement at 77 K for **CPO-27-NCS**, closed symbols: adsorption, open symbols: desorption. 10

Figure S5. Ar adsorption measurement at 87 K for **CPO-27(Mg)-NCS**, closed symbols: adsorption, open symbols: desorption. 11

Figure S6. IR spectra of the as-synthesized **CPO-27** immersed in solvents (ethanol, methanol and dimethyloformamide) containing dissolved NH_4SCN. The IR spectra of **CPO-27-NCS** are given for comparison. 11

Figure S7. IR spectra (left) and PXRD patterns (right) of the as-synthesized **CPO-27** immersed in saturated solutions of NH_4SCN in ethanol. The IR spectra and PXRD patterns of **CPO-27-NCS** are given for comparison. PXRD patterns: **CPO-27** calculated from SC-XRD data (green), **CPO-27-NCS** calculated from DFT+D optimized structures (black) 12

Figure S8. Reversibility of **CPO-27-NCS** formation: IR spectra (left) and PXRD patterns (right): **CPO-27** as-synthesized (black), **CPO-27-NCS** (red) and **CPO-27-NCS** immersed in methanol at 60°C for 5 days - **CPO-27-NCS** MeOH (green). 13

Figure S9. TG (black) and dTG (red) curves for **CPO-27-NCS** showing stepwise weight loss upon heating. 14

Table S1. Calculated scaled wavenumbers, ν (in cm^{-1}) with CN bond lengths (in Å) for the modified **CPO-27** materials compared with experimental wavenumbers (exp). 14

Figure S10. PXRD patterns: **CPO-27** calculated from SC-XRD data (green), **CPO-27-NCS** calculated from DFT+D optimized structures (black), and **CPO-27-NCS** recorded for the as-synthesized bulk material (red).. 14

Figure S11. IR spectra (top), optical images (center) and PXRD patterns (bottom) of mixtures after grinding **CPO-27(Co)** with NH_4SCN at the 6:3 **CPO-27** to NH_4SCN ratio. For comparison, IR spectra, optical image and PXRD pattern of the $[Co(SCN)_4]^{2-}$ ions, are given. 15

Figure S12. Variable temperature EIS conductivity measurements at 30% RH for **CPO-27(Mg)-NCS, CPO-27(Zn)-NCS** and **CPO-27(Ni)-NCS**. 15

Figure S13. Variable temperature EIS conductivity measurements at 45% RH for **CPO-27(Mg)-NCS**, **CPO-27(Zn)-NCS** and **CPO-27(Ni)-NCS**. 16

Figure S14. Variable temperature EIS conductivity measurements at 60% RH for **CPO-27(Mg)-NCS**, **CPO-27(Zn)-NCS** and **CPO-27(Ni)-NCS**. 16

Figure S15. Variable temperature EIS conductivity measurements at 75% RH for **CPO-27(Mg)-NCS**, **CPO-27(Zn)-NCS** and **CPO-27(Ni)-NCS**. 16

Figure S16. Variable temperature EIS conductivity measurements at 90% RH for **CPO-27(Mg)-NCS**, **CPO-27(Zn)-NCS** and **CPO-27(Ni)-NCS**. 16

Figure S17. Arrhenius plots with activation energies indicated as numbers (in eV) for various RH values (top legend) for **CPO-27-NCS**.. 17

Figure S18. Proton conductivity at different RH and temperatures for **CPO-27(Mg)-NCS**, **CPO-27(Zn)-NCS** and **CPO-27(Ni)-NCS**. 17

Figure S19. Variable temperature EIS conductivity measurements at 90% RH for **CPO-27(Mg)-NCS** with various stoichiometric ratios: 6:1, 6:2, 6:3 (**CPO-27** to NH_4SCN). 17

Figure S20. Variable temperature EIS conductivity measurements at 90% RH for **CPO-27(Ni)-NCS** with various stoichiometric ratios: 6:1, 6:2, 6:3 (**CPO-27** to NH_4SCN)... 17

Figure S21. Variable temperature EIS conductivity measurements at 90% RH for **CPO-27(Zn)-NCS** with various stoichiometric ratios: 6:1, 6:2, 6:3 (**CPO-27** to NH_4SCN).. 18

Figure S22. Arrhenius plots with activation energies indicated as numbers (in eV) at 90% RH for **CPO-27-NCS** with various stoichiometric amounts of NH_4SCN added; given as 6:1, 6:2, 6:3 **CPO-27** to NH_4SCN ratios. 18

Figure S23. Proton conductivity change with temperature at 90% RH for **CPO-27-NCS** with various stoichiometric amounts of NH_4SCN added; given as 6:1, 6:2, 6:3 **CPO-27** to NH_4SCN ratios.. 18

Table S2. Comparison of proton conductivities for **CPO-27-NCS**................................ 19

Table S3. Comparison of proton conductivities for **CPO-27(Mg)-NCS** with various with various stoichiometric amounts of NH_4SCN added; given as 6:1, 6:2, 6:3 **CPO-27** to NH_4SCN ratios. 19

Table S4. Comparison of proton conductivities for **CPO-27(Ni)-NCS** with various with various stoichiometric amounts of NH_4SCN added; given as 6:1, 6:2, 6:3 **CPO-27** to NH_4SCN ratios. 19

Table S5. Comparison of proton conductivities for **CPO-27(Zn)-NCS** with various with various stoichiometric amounts of NH_4SCN added; given as 6:1, 6:2, 6:3 **CPO-27** to NH_4SCN ratios. 19

Table S6. Proton conductivities for selected MOFs including those with protonated amines/ammonia and those based on **CPO-27**(**MOF-74**) materials. 20

Figure S24. H_2O adsorption cycling test for **CPO-27-NCS**. 21

Figure S25. H_2O vapor adsorption measurement at 298 K for **CPO-27-NCS**, closed symbols: adsorption, open symbols desorption. 21

Figure S26. IR spectra (left) and PXRD patterns (right) of the as-synthesized **CPO-27-NCS** compared with those of samples conditioned at 90% RH before EIS measurement (red) and after EIS measurement (green). 22

Figure S27. Representative temperature-dependent AC impedance plots at 45% RH for **CPO-27(Zn)-NCS** (numbers indicate temperatures in ^{o}C) with the equivalent circuit used for fitting the plots (ZSimpWin software). 23

References in the Supporting Information 23

Synthetic procedures and control experiments

All chemicals and solvents (of analytical grade) were purchased from commercial sources (Merck, ,TCI, Avantor, Polmos) and were used without further purification. Ethanol (Polmos) contained water (8% by volume).

Synthesis of ***CPO-27(Zn)***

CPO-27(Zn) was synthesized according to a method described in literature.[1] H_4DOBC (0.25 g, 1.3 mmol) and $Zn(NO_3)_2{\cdot}4H_2O$ (1.0 g, 3.8 mmol) were dissolved in 50 mL of DMF with stirring, followed by the addition of 2.5 mL of deionized water. The mixture was heated in an oven at 100°C for 20 hours yielding the yellow microcrystalline, porous material, **CPO-27(Zn)**.

Synthesis of ***CPO-27(Mg)***

CPO-27(Mg) was synthesized according to a method described in literature.[2] To a solid mixture of H_4DOBDC (0.111 g, 0.559 mmol) and $Mg(NO_3)_2{\cdot}6H_2O$ (0.475 g, 1.85 mmol) was added a 15:1:1 (v/v/v) mixture of DMF–ethanol–water (50 mL) in a 100 mL screw cap jar. The suspension was mixed and ultrasonicated until homogeneous and placed in an oven at 100°C. After 20 hours, the samples were removed from the oven and allowed to cool to RT. The mother liquor was decanted from the yellow microcrystalline material and replaced with methanol. The methanol was decanted and replenished four times over two days, yielding the dark yellow microcrystalline, porous material.

Synthesis of ***CPO-27(Ni)***

CPO-27(Ni) was synthesized according to a method described in literature.[2] To a solid mixture of H_4DOBDC (0.0956 g, 0.48 mmol) and $Ni(NO_3)_2{\cdot}6H_2O$ (0.4756 g, 1.64 mmol) was added a 1:1:1 (v/v/v) mixture of DMF–ethanol–water (40 mL) in a 100 mL screw cap jar. The suspension was mixed and ultrasonicated until homogeneous and placed in an oven at 100°C. After 20 hours, the samples were removed from the oven and allowed to cool to RT. The mother liquor was decanted from the yellow microcrystalline material and replaced with methanol. The methanol was decanted and replenished four times over two days, yielding the yellow-brown microcrystalline, porous material.

Synthesis of ***CPO-27(Co)***

CPO-27(Co) was synthesized according to a method described in literature.[2] To a solid mixture of H_4DOBDC (0.0964 g, 0.47 mmol) and $Co(NO_3)_2{\cdot}6H_2O$ (0.4754 g, 1.73 mmol) was added a 1:1:1 (v/v/v) mixture of DMF–ethanol–water (40 mL) in a 100 mL screw cap jar. The suspension was mixed and ultrasonicated until homogeneous and placed in an oven at 100°C. After 20 hours, the samples were removed from the oven and allowed to cool to RT. The mother liquor was decanted from the dark red-purple microcrystalline material and replaced with methanol. The methanol was decanted and replenished four times over two days, yielding the dark red-purple microcrystalline, porous material.

Synthesis of ***CPO-27(Zn)-NCS*** - $\{(NH_4)_3[Zn_6(dobdc)_3(NCS)_3(dmf)(H_2O)_2]{\cdot}xH_2O\}$

CPO-27(Zn) (100.0 mg, 0.197 mmol) and NH_4SCN (15.0 mg, 0.197 mmol) were ground in an agate mortar in air at room temperature for approx. 10 min. with addition of 57.5 μL 92%

EtOH (LAG, liquid-assisted grinding; 6:3 metal/thiocyanate ratio). Elem. anal.: Calc. for $C_{30}H_{43}Zn_6N_7O_{28}S_3$ ({$(NH_4)_3[Zn_6(dobdc)_3(NCS)_3(dmf)(H_2O)_2]\cdot 7H_2O$}): C 24.75, H 3.46, N 6.87, S 6.29%. Found: C 24.20, H 3.08, N 6.23, S 7.10%. IR (ATR, cm^{-1}): ν(COO_{as}) 1549vs, ν(COO_s) 1402s, ν$(CO)_{DMF}$ 1649s, ν$(CN)_{SCN}$ 2089vs, ν$(NH)_{ammonium}$ 3182m. To synthesize **CPO-27(Zn)-NCS** at lower stoichiometric ratios (6:1 and 6:2), the amounts of NH_4SCN were reduced to 5 mg and 10 mg, and the amount of EtOH used was 52.5 μL and 55 μL, respectively.

Synthesis of ***CPO-27(Mg)-NCS*** - {$(NH_4)_3[Mg_6(dobdc)_3(NCS)_3(H_2O)_3]\cdot xH_2O$}
CPO-27(Mg) (100.0 mg, 0.292 mmol) and NH_4SCN (22.2 mg, 0.292 mmol) were ground in an agate mortar in air at room temperature for approx. 10 min. with addition 61.1 μL 92% EtOH (LAG, liquid-assisted grinding; 6:3 metal/thiocyanate ratio). Elem. anal.: Calc. for $C_{27}H_{34}Mg_6N_6O_{26}S_3$ ({$(NH_4)_3[Mg_6(dobdc)_3(NCS)_3(H_2O)_3]\cdot 5H_2O$}): C 29.46, H 3.11, N 7.64, S 8.74%. Found: C 28.52, H 3.84, N 8.68, S 8.60%. IR (ATR, cm^{-1}): ν(COO_{as}) 1575vs, ν(COO_s) 1417s, ν$(CN)_{SCN}$ 2078vs, ν$(NH)_{ammonium}$ 3221m. To synthesize **CPO-27(Mg)-NCS** at lower stoichiometric ratios (6:1 and 6:2), the amounts of NH_4SCN were reduced to 7.4 mg and 14.8 mg, and the amount of EtOH used was 53.7 μL and 57.4 μL, respectively.

Synthesis of ***CPO-27(Ni)-NCS*** - {$(NH_4)_3[Ni_6(dobdc)_3(NCS)_3(H_2O)_3]\cdot xH_2O$}
CPO-27(Ni) (100.0 mg, 0.254 mmol) and NH_4SCN (19.2 mg, 0.254 mmol) were ground in an agate mortar in air at room temperature for approx. 10 min. with addition 59.6 μL 92% EtOH (LAG, liquid-assisted grinding; 6:3 metal/thiocyanate ratio). Elem. anal.: Calc. for $C_{27}H_{42}Ni_6N_6O_{30}S_3$ ({$(NH_4)_3[Ni_6(dobdc)_3(NCS)_3(H_2O)_3]\cdot 9H_2O$}): C 23.52, H 3.07, N 6.09, S 6.98%. Found: C 23.21, H 3.18, N 7.09, S 6.98%. IR (ATR, cm^{-1}): ν(COO_{as}) 1548vs, ν(COO_s) 1404s, ν$(CN)_{SCN}$ 2101vs, ν$(NH)_{ammonium}$ 3206m. To synthesize **CPO-27(Ni)-NCS** at lower stoichiometric ratios (6:1 and 6:2), the amounts of NH_4SCN were reduced to 6.4 mg and 12.8 mg, and the amount of EtOH used was 53.2 μL and 56.4 μL 92%, respectively.

Control experiments to test the possibility of formation of **CPO-27-NCS** in solution
Several trials described below have been carried out with various solvents. In all experiments **CPO-27** (100 mg) and NH_4SCN (at 1:10 **CPO-27** to NH_4SCN ratio) were mixed in a solvent (10.0 mL) and left for approx. 72h at room temperature.
1) **CPO-27(Mg)**; NH_4SCN (74 mg); ethanol or methanol
2) **CPO-27(Ni)**; NH_4SCN (64 mg); ethanol or methanol
3) **CPO-27(Zn)**; NH_4SCN (50 mg); ethanol or dimethyloformamide
Analogous trials were also performed for saturated alcohol solutions of NH_4SCN. All solid samples have been screened with IR (Figures S6 and S7 below). Quantitative formation of **CPO-27(Mg, Zn Ni)-NCS** was not formed.

Reversibility of **CPO-27-NCS** formation
In all experiments **CPO-27-NCS** (50 mg) were immersed in methanol (5.0 mL) for approx. 5 days at 60°C. During this time methanol was changed 4 times a day.
All solid samples have been screened with IR and PXRD (Figure S8 below). In all the trials **CPO-27(Mg, Zn, Ni)** were recovered.
Analogous trials carried out in methanol at approx. 25°C did not lead to removal of coordinated thiocyanate after 7 days.

Samples preparation for EIS measurements

Prior to a series of EIS measurements at a given relative humidity (RH), approx. 30 mg of the material was equilibrated in a humidity chamber for 1 day at a specified RH (90 or 75 or 60 or 45 or 30%) at 25°C.

Details of physical measurements

Carbon, hydrogen and nitrogen were determined using an Elementar Vario MICRO Cube elemental analyzer.

Thermogravimetric analyses (TGA) were performed on a Mettler-Toledo TGA/SDTA 851^e instrument with a heating rate of 10°C min^{-1} in a temperature range of 25 – 600°C (approx. sample weight of 10 mg). The measurements were performed at atmospheric pressure under argon flow.

FTIR spectra were recorded on a Thermo Scientific Nicolet iS10 FT-IR spectrophotometer equipped with an iD7 diamond ATR attachment.

Powder X-ray diffraction (PXRD) patterns were recorded at room temperature (295 K) on a Rigaku Miniflex 600 diffractometer with Cu-Kα radiation (λ = 1.5418 Å) in a 2θ range from 3° to 45° with a 0.02° step and 3° min^{-1} scan speed. Additional measurement was carried out for **CPO-27(Mg)-NCS** on a Panalytical X-Pert Pro equipped with a sealed LFF Cu X-ray source operating at 40kV and 30 mA, a focusing mirror, a capillary sample holder and a PIXCEL detector.

Electrochemical Impedance Spectroscopy (EIS) measurements were performed using Hioki IM3570 impedance analyzer on pre-conditioned samples (see Samples preparation above) pressed between two metallic electrodes (5.0 mm diameter) in a PTFE tube, and kept in KK 115 TOP+ or Memmert HCP 246 climatic chamber with ultrasonic humidifier and temperature control. The measurements were carried out in a quasi-four-probe setup in a frequency range from 4 Hz to 5MHz, alternating potential of 200 mV and temperature range from 25 to 60 °C (for 30-90% RH), and with pre-measurement sample conditioning for 1 h at each temperature. The proton conductivity (σ, S cm^{-1}) of the sample was estimated by using the equation:

$$\sigma = L/(RA)$$

where L (cm) is the sample thickness and A (cm^2) is the cross-sectional area of the measured pellet; R (Ω) is the resistance as the real part of the measured impedance.

Adsorption isotherms were measured using static volumetric Autosorb IQ apparatus (Quantachrome Instruments) at 77 K for nitrogen and 298 K for water. All samples were activated under vacuum for 8 h at 100°C (2°C/min).

Computational details

All the quantum-chemical calculations of energies, geometries, and vibrational analyses were performed by using of DMol3[3] code at the periodic DFT level of theory, the RPBE[4,5] correlation-exchange functional. This functional was chosen since it provides a better description of the hydrogen bonds than PBE or revPBE. The orbitals were expanded in the

basis set of DND quality. The basis set spatial cutoff distance of 4.2 Å was set and, as a convergence accelerator, the Gaussian smearing with the window width of $5 \cdot 10^{-3}$ Ha was used. The SCF convergence criterion was set to $1 \cdot 10^{-6}$ Ha, whereas for geometry optimisation – to the threshold value of $2 \cdot 10^{-5}$ Ha change between two successive iterations, the gradient norm lower than $3 \cdot 10^{-3}$ Ha⁄Å, and the maximal atom displacement lower than $4 \cdot 10^{-3}$ Å.

Rietveld refinement procedure

The X-ray diffraction of the **CPO-27(Mg)-NCS** sample has been performed in a long collection time capillary experiment and a trial has been undertaken to determine the crystal structure of **CPO-27(Mg)-NCS** through the application of Rietveld refinement using the published structure as the starting model (CSD code: MOHGOI). Given the space group (R-3) of the initial **CPO-27(Mg)** structure, the insertion of NCS^- and NH_4^+ ions results in their six-fold replication. We have tried to refine this model with 50% site occupancies which led us to problematic electron density from NH_4^+ ions which caused them to drift towards the centre of the main [001] channel (resulting distances between nitrogen atoms were not physical). At the same time, the NCS^- ions were clearly visible in the Fourier maps in the proper locations consistent with the theoretical calculations. We have also tried lowering the symmetry of the loaded structure to R3 to overcome the problem introduced by the six-fold replication inside the channel (the XRD patterns are undistinguishable for R-3 and R3 apart from the intensity statistics that cannot be reliably performed for powder data) but it did not lead to a significant improvement. Most likely our data (laboratory X-ray source) are of insufficient quality to unambiguously locate the positions of NH_4^+ and SCN^- ions. Also, there is a significant possibility of disorder in terms of their positions since there is no difference between the Mg sites that would make some of them preferential over the others. It is noteworthy that in the original work reporting MOHGOI structure[6] the authors used synchrotron radiation which provided powder X-ray diffraction data with excellent signal-to-noise ratios and allowed crystallographic studies with high level of detail, even including the localization of the adsorbed CO_2 molecules.

Figures and Tables

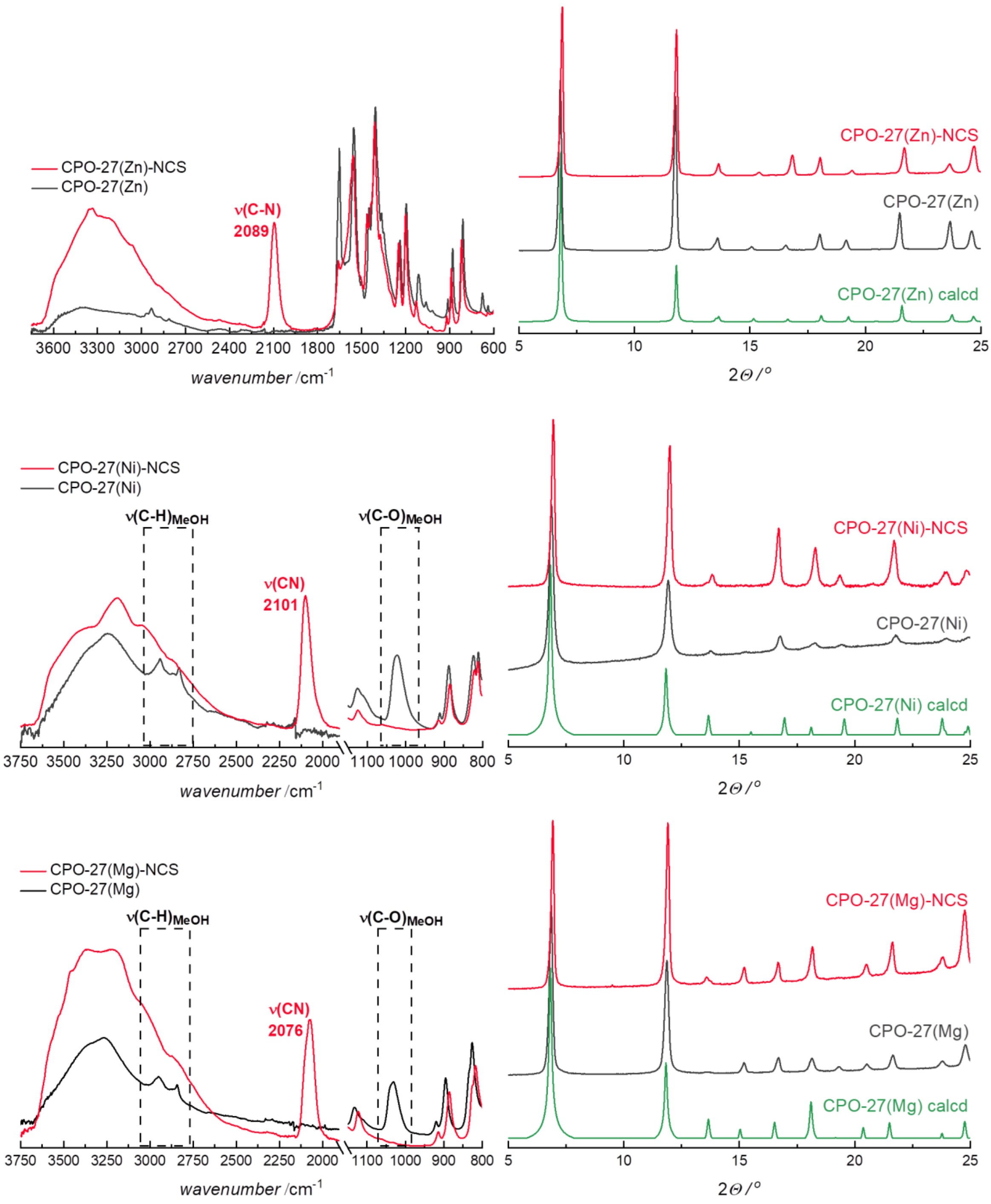

Figure S1. IR spectra (left): **CPO-27** as-synthesized (black) and **CPO-27(M)-NCS** (**CPO-27** after LAG with NH_4SCN at 6:3 molar ratio (red). PXRD patterns (right): **CPO-27** calculated from SC-XRD data (green), the as-synthesized bulk material (black), and **CPO-27(M)-NCS** (red).

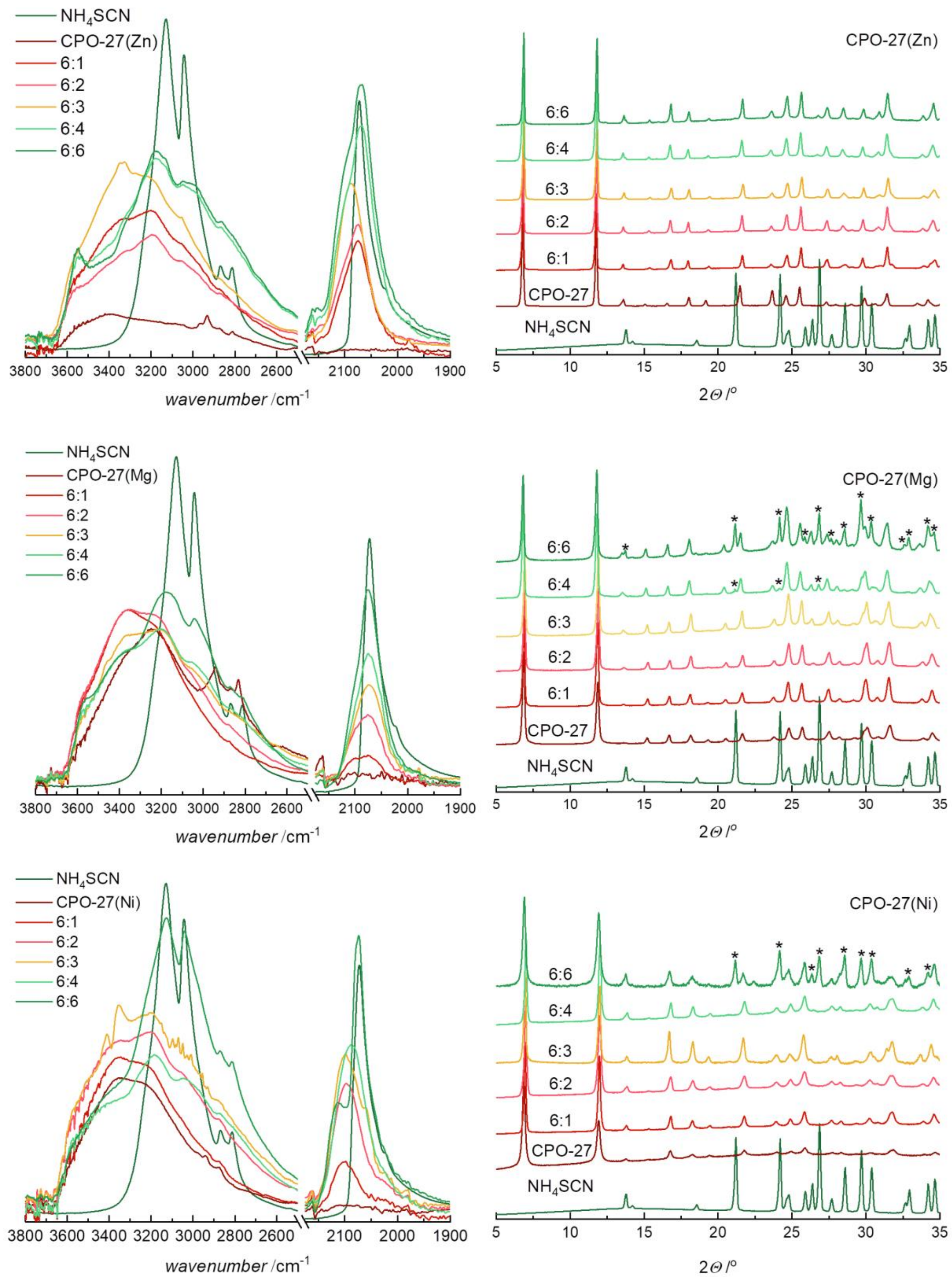

Figure S2. IR spectra (left) and PXRD patterns (right) of mixtures after grinding **CPO-27** with various amounts of NH_4SCN; 6:1, 6:2, 6:3, 6:4, 6:6 (given as **CPO-27** to NH_4SCN ratio). Characteristic reflections of NH_4SCN are labeled with '*'.

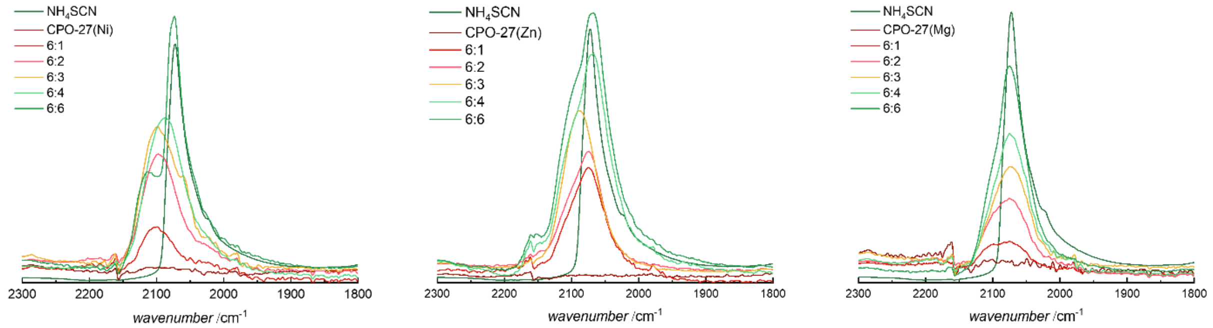

Figure S3. IR spectra of mixtures after grinding **CPO-27** with various amounts of NH_4SCN; 6:1, 6:2, 6:3, 6:4, 6:6 (given as the **CPO-27** to NH_4SCN ratio) showing v(CN) range.

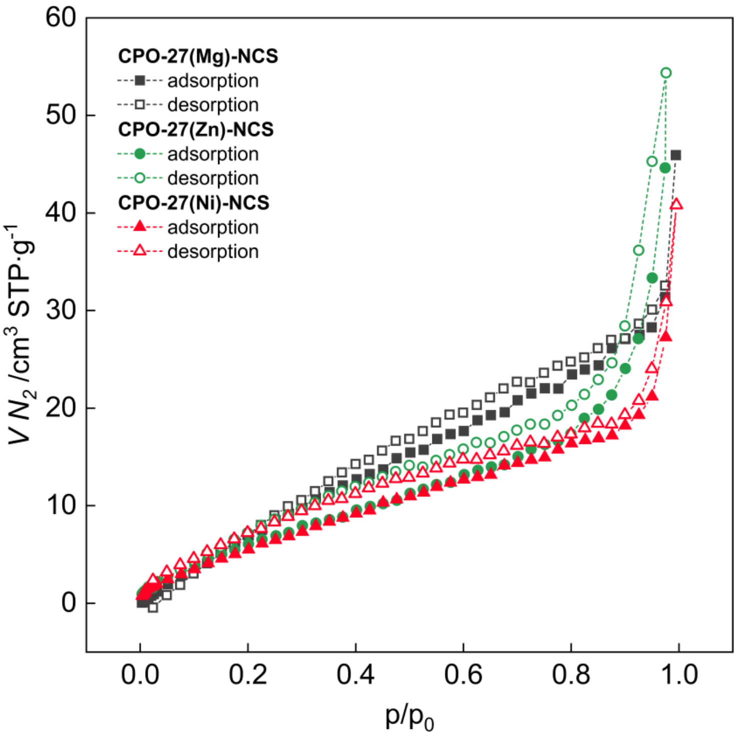

Figure S4. N_2 adsorption measurement at 77 K for **CPO-27-NCS**, closed symbols: adsorption, open symbols: desorption.

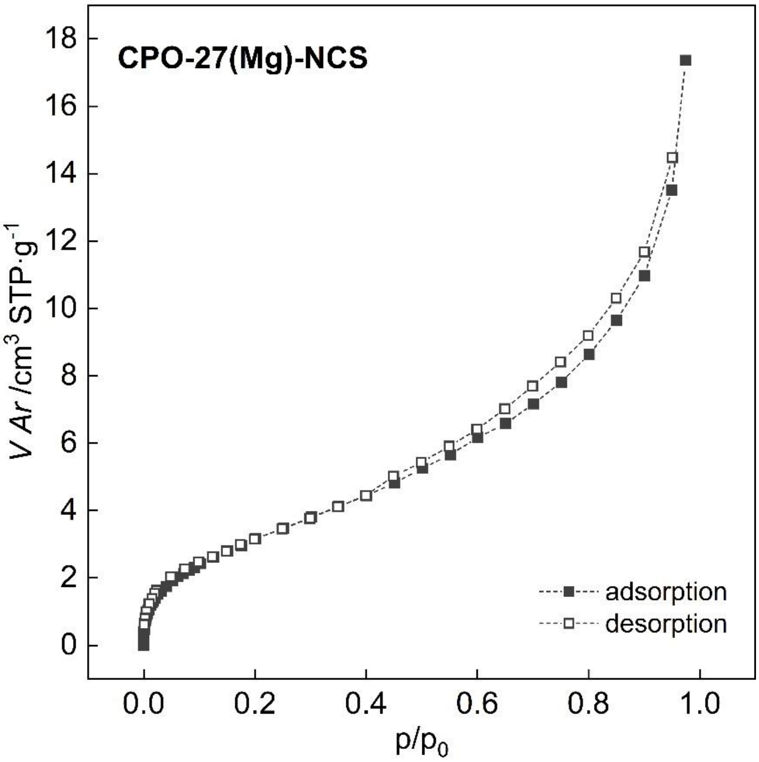

Figure S5. Ar adsorption measurement at 87 K for **CPO-27(Mg)-NCS**, closed symbols: adsorption, open symbols: desorption.

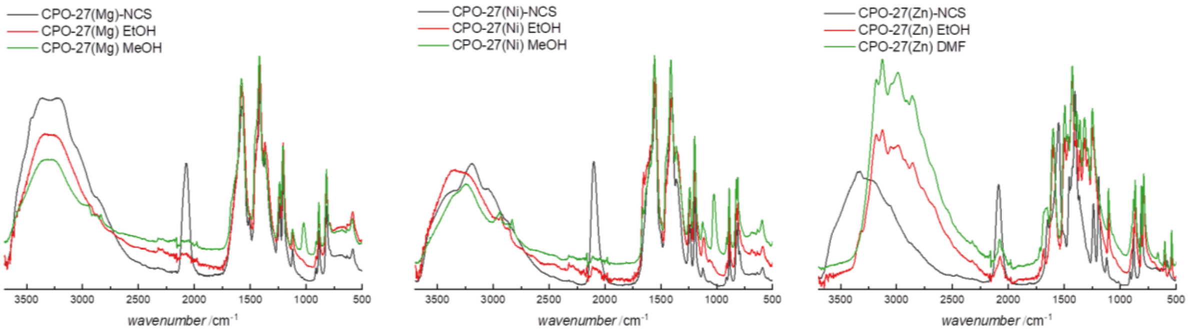

Figure S6. IR spectra of the as-synthesized **CPO-27** immersed in solvents (ethanol, methanol and dimethyloformamide) containing dissolved NH_4SCN. The IR spectra of **CPO-27-NCS** are given for comparison.

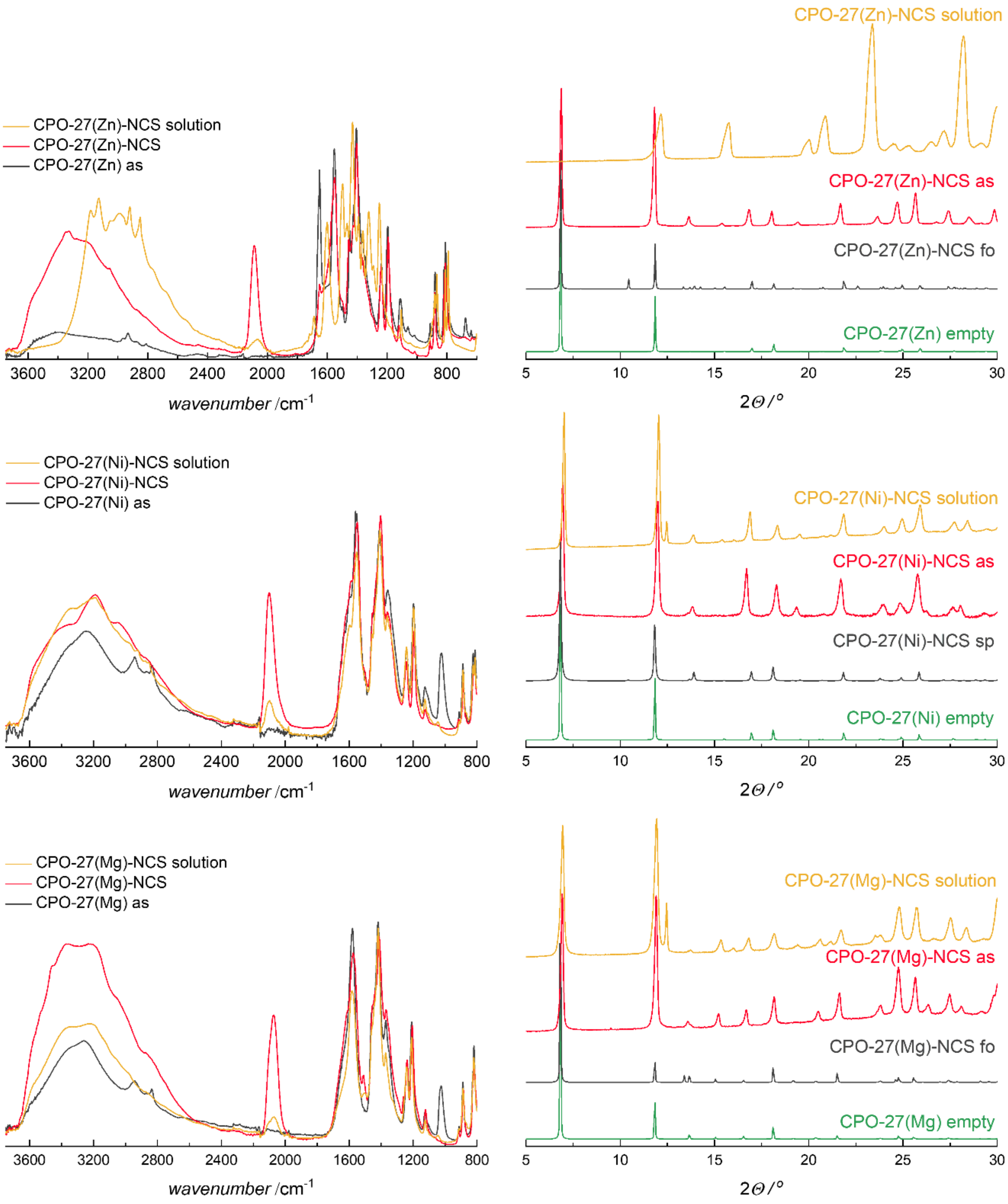

Figure S7. IR spectra (left) and PXRD patterns (right) of the as-synthesized **CPO-27** immersed in saturated solutions of NH_4SCN in ethanol. The IR spectra and PXRD patterns of **CPO-27-NCS** are given for comparison. PXRD patterns: **CPO-27** calculated from SC-XRD data (green), **CPO-27-NCS** calculated from DFT+D optimized structures (black).

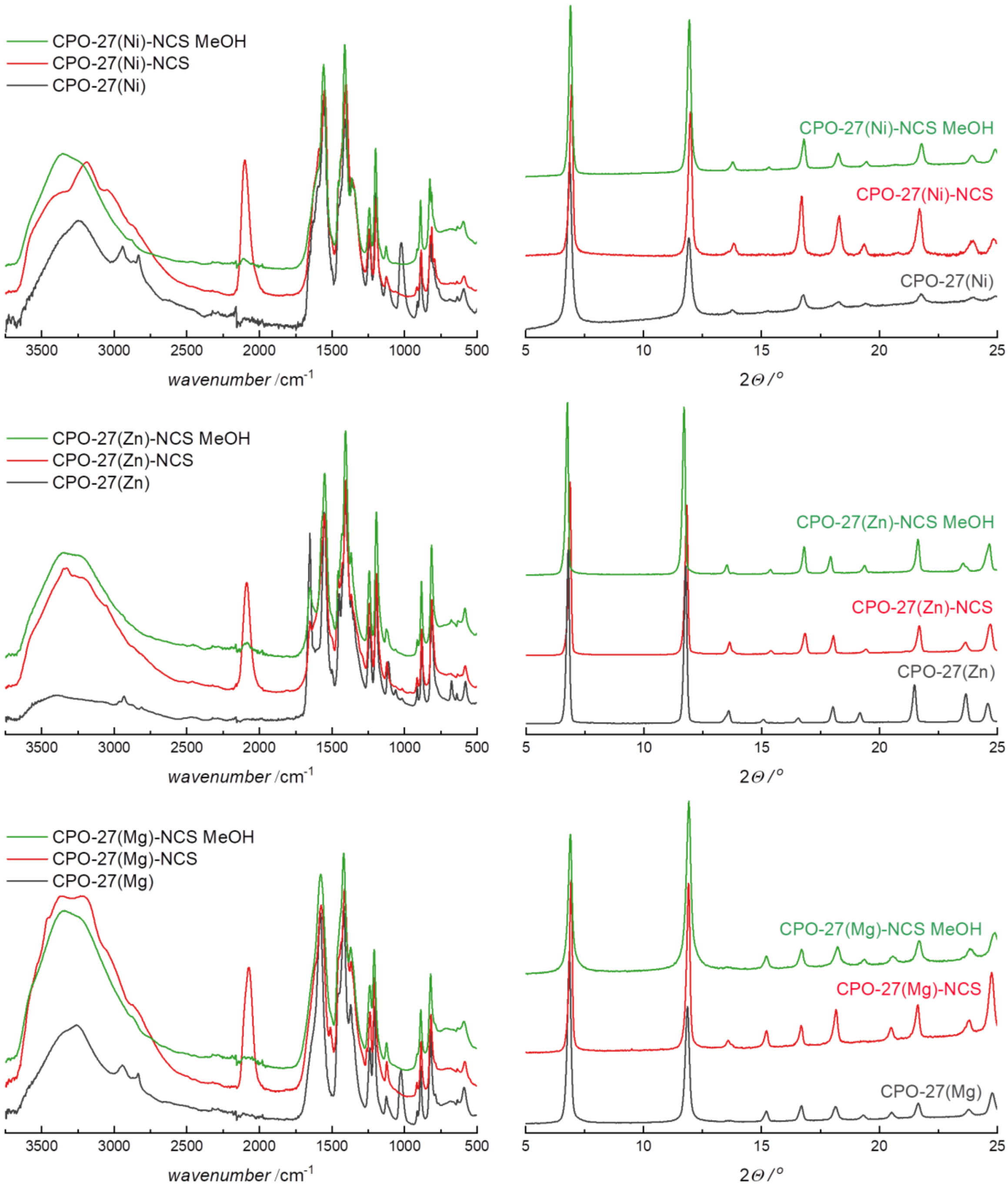

Figure S8. Reversibility of **CPO-27-NCS** formation: IR spectra (left) and PXRD patterns (right): **CPO-27** as-synthesized (black), **CPO-27-NCS** (red) and **CPO-27-NCS** immersed in methanol at 60°C for 5 days - **CPO-27-NCS** MeOH (green).

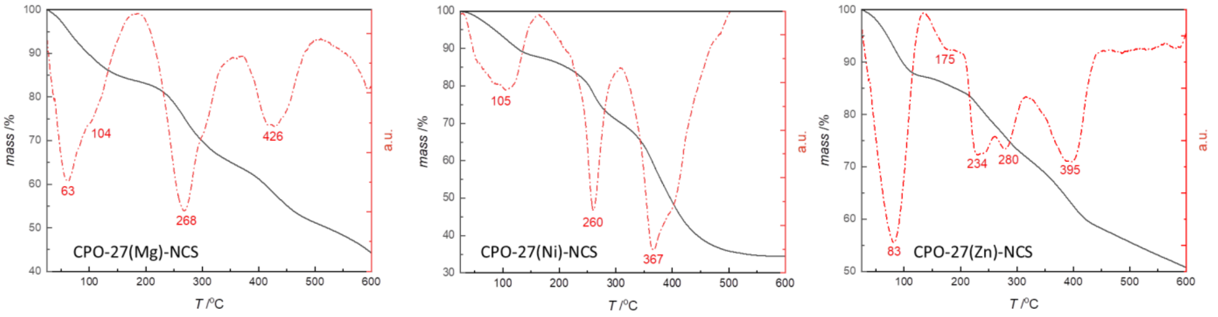

Figure S9. TG (black) and dTG (red) curves for **CPO-27-NCS** showing stepwise weight loss upon heating.

Table S1. Calculated scaled wavenumbers, *v* (in cm^{-1}) with CN bond lengths (in Å) for the modified **CPO-27** materials compared with experimental wavenumbers (exp).

Sample		Mg^{2+}	Zn^{2+}	Ni^{2+} (*)
Calc. M-SCN	*v*, cm^{-1}	1983	2064	(1996)
	d(CN)	1,202	1,186	1,200
Calc. M-NCS	*v*, cm^{-1}	2078	2096	(2079)
	d(CN)	1,184	1,184	1,185
Exp.	*v*, cm^{-1}	2076	2089	2101

* values given in parentheses are estimated on the basis of CN bond length

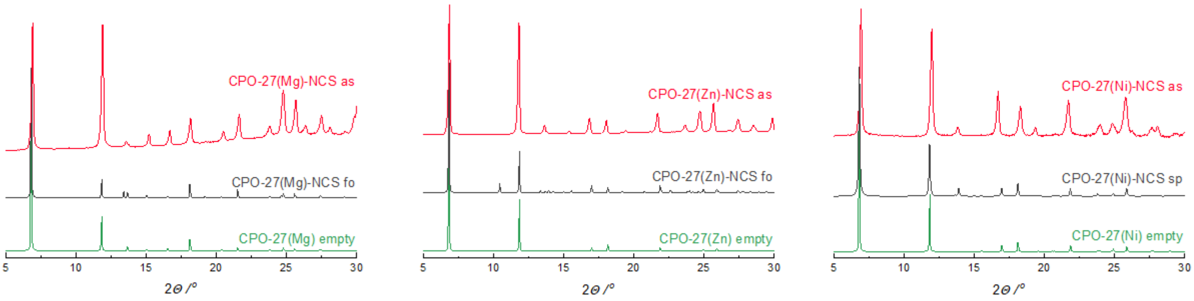

Figure S10. PXRD patterns: **CPO-27** calculated from SC-XRD data (green), **CPO-27-NCS** calculated from DFT+D optimized structures (black), and **CPO-27-NCS** recorded for the as-synthesized bulk material (red).

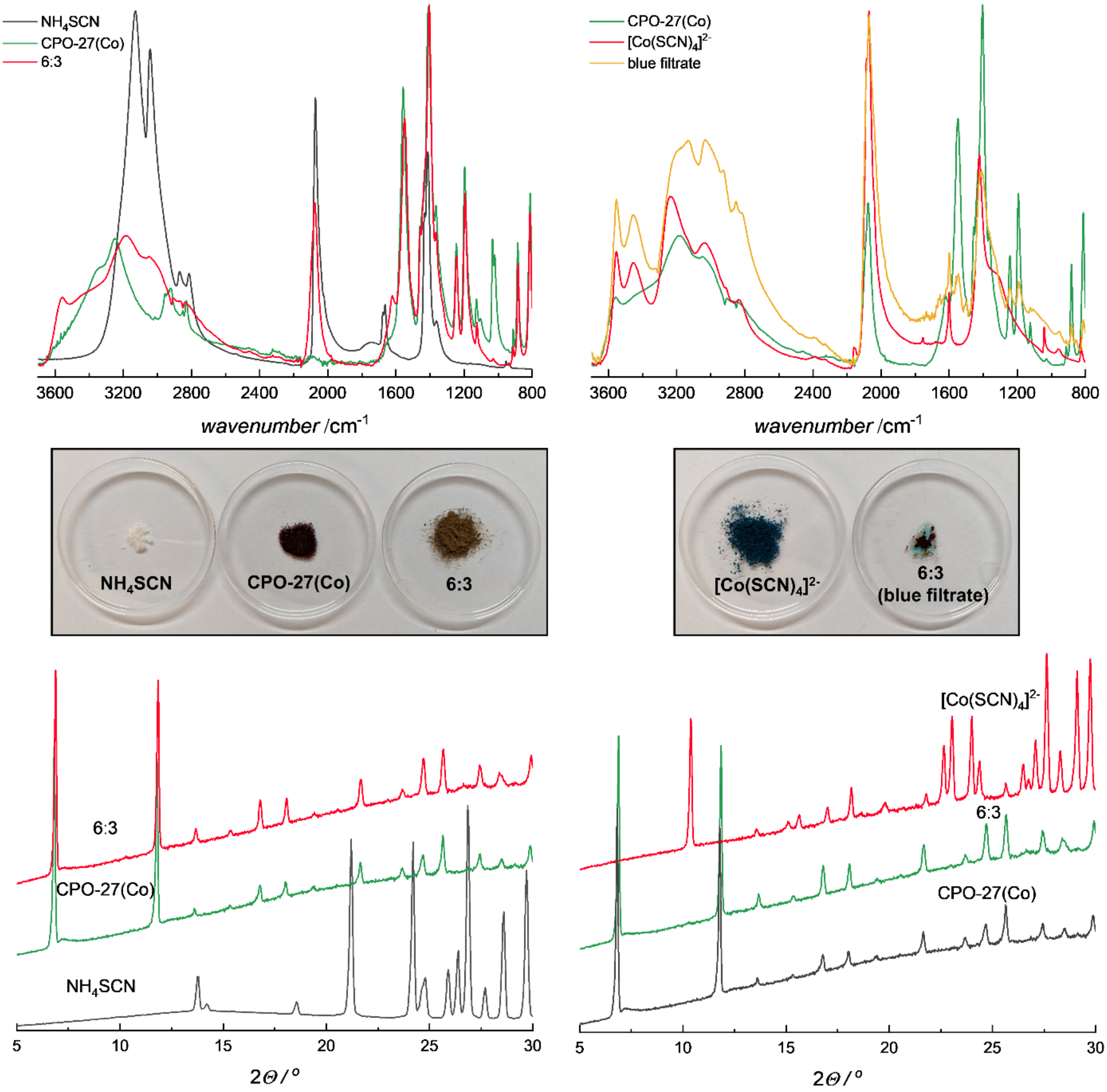

Figure S11. IR spectra (top), optical images (center) and PXRD patterns (bottom) of mixtures after grinding **CPO-27(Co)** with NH_4SCN at the 6:3 **CPO-27** to NH_4SCN ratio. For comparison, IR spectra, optical image and PXRD pattern of the $[Co(SCN)_4]^{2-}$ ions, are given.

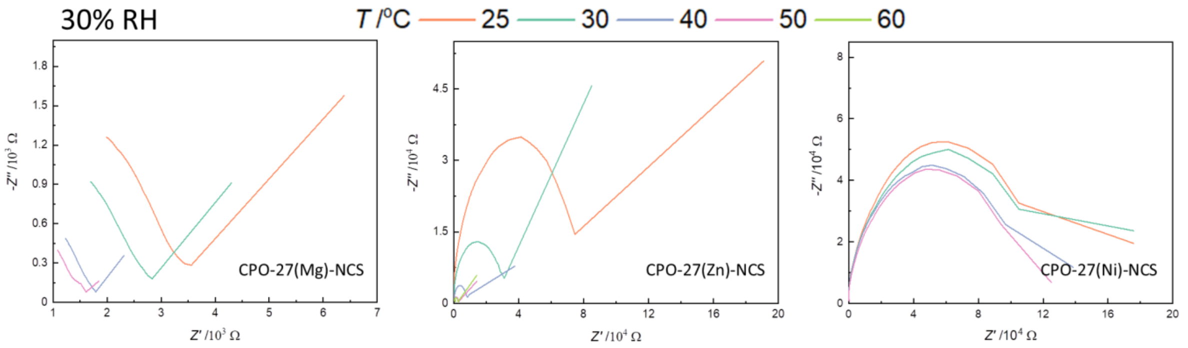

Figure S12. Variable temperature EIS conductivity measurements at 30% RH for **CPO-27(Mg)-NCS**, **CPO-27(Zn)-NCS** and **CPO-27(Ni)-NCS**.

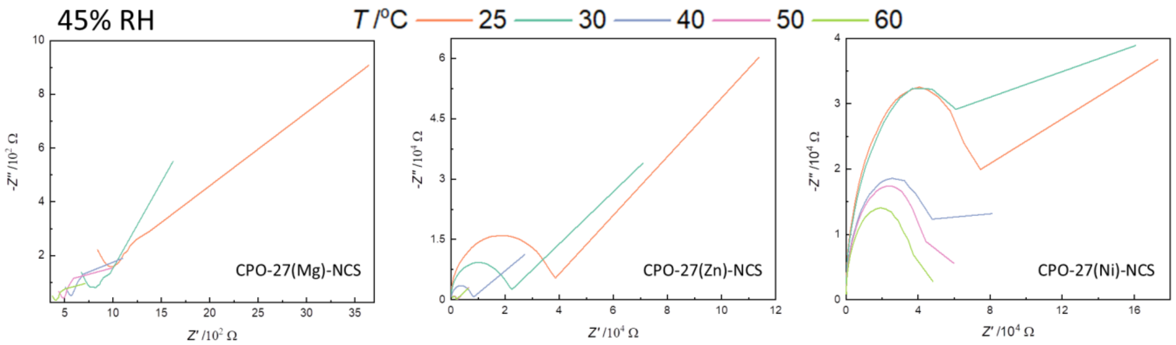

Figure S13. Variable temperature EIS conductivity measurements at 45% RH for **CPO-27(Mg)-NCS**, **CPO-27(Zn)-NCS** and **CPO-27(Ni)-NCS**.

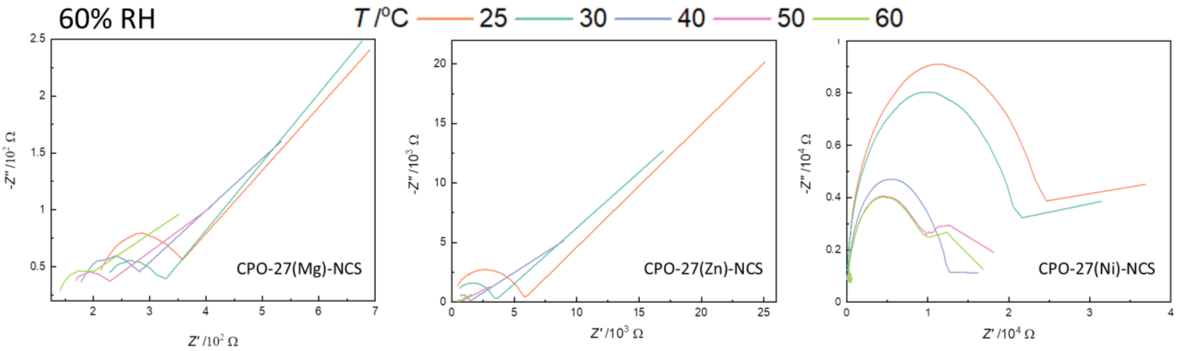

Figure S14. Variable temperature EIS conductivity measurements at 60% RH for **CPO-27(Mg)-NCS**, **CPO-27(Zn)-NCS** and **CPO-27(Ni)-NCS**.

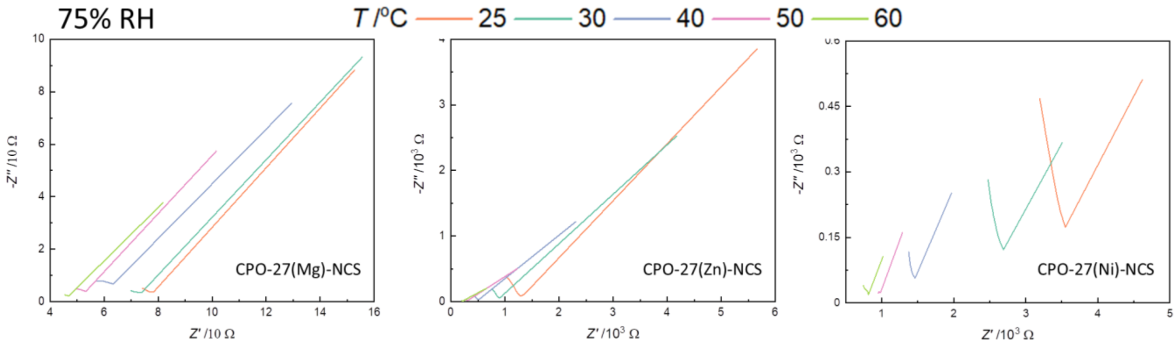

Figure S15. Variable temperature EIS conductivity measurements at 75% RH for **CPO-27(Mg)-NCS**, **CPO-27(Zn)-NCS** and **CPO-27(Ni)-NCS**.

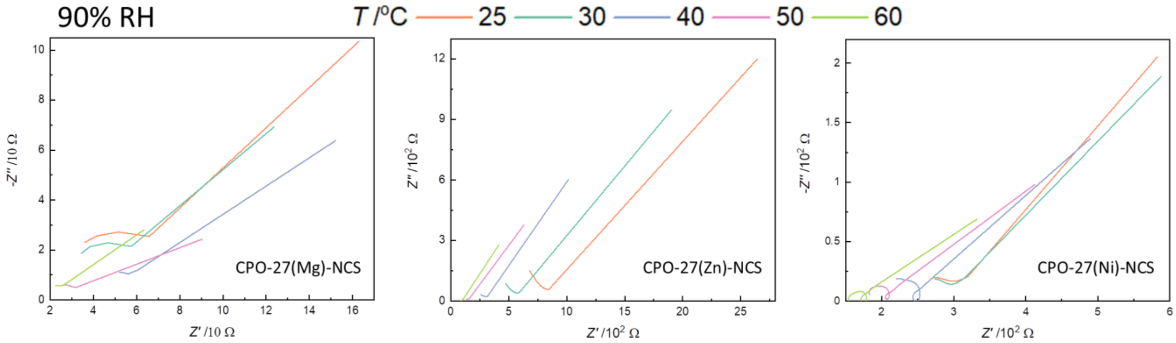

Figure S16. Variable temperature EIS conductivity measurements at 90% RH for **CPO-27(Mg)-NCS**, **CPO-27(Zn)-NCS** and **CPO-27(Ni)-NCS**.

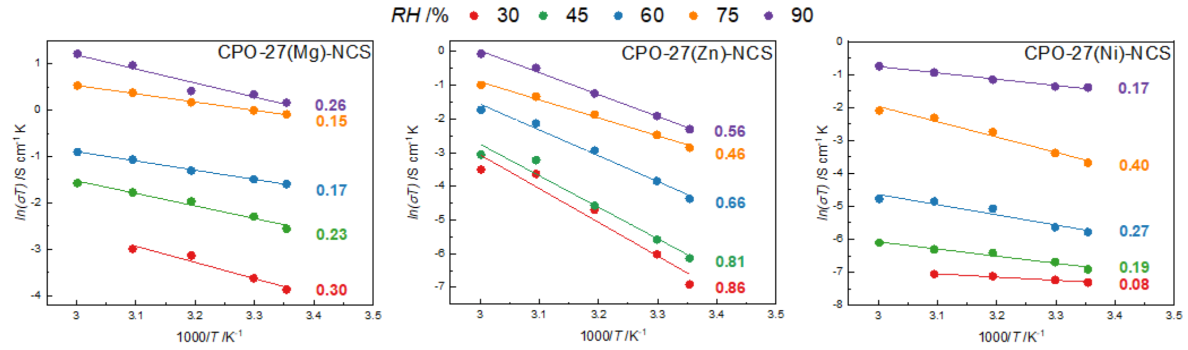

Figure S17. Arrhenius plots with activation energies indicated as numbers (in eV) for various RH values (top legend) for **CPO-27-NCS**.

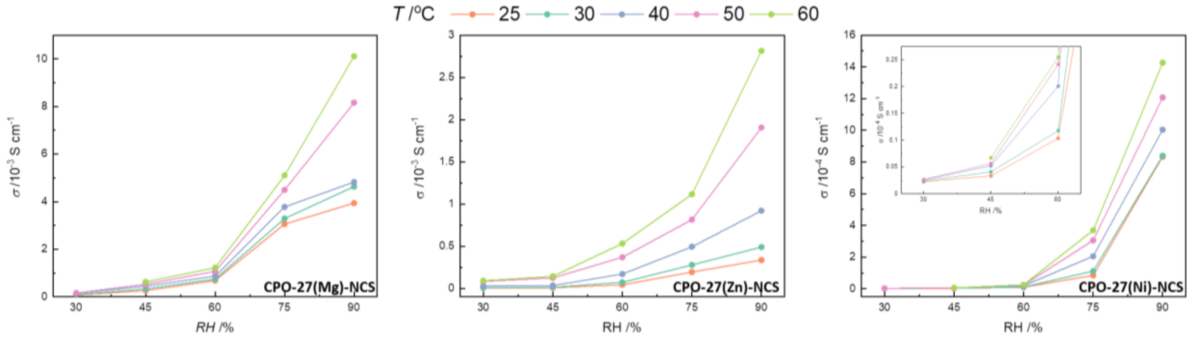

Figure S18. Proton conductivity at different RH and temperatures for **CPO-27(Mg)-NCS**, **CPO-27(Zn)-NCS** and **CPO-27(Ni)-NCS**.

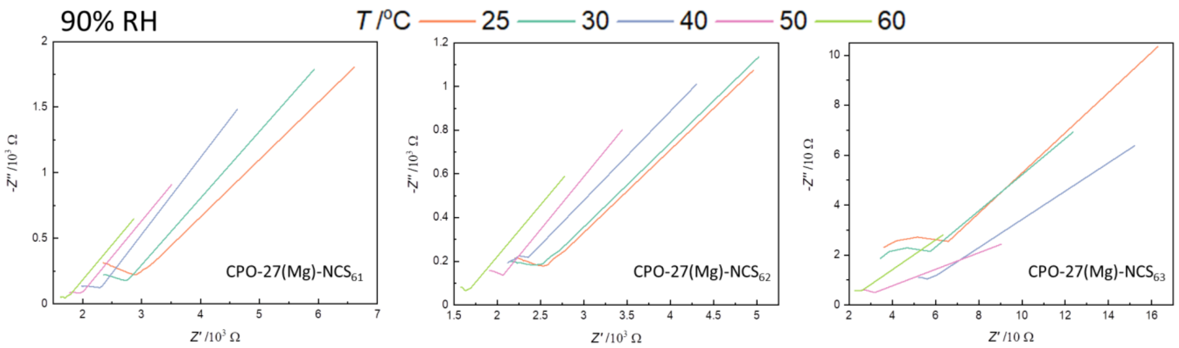

Figure S19. Variable temperature EIS conductivity measurements at 90% RH for **CPO-27(Mg)-NCS** with various stoichiometric ratios: 6:1, 6:2, 6:3 (**CPO-27** to NH_4SCN).

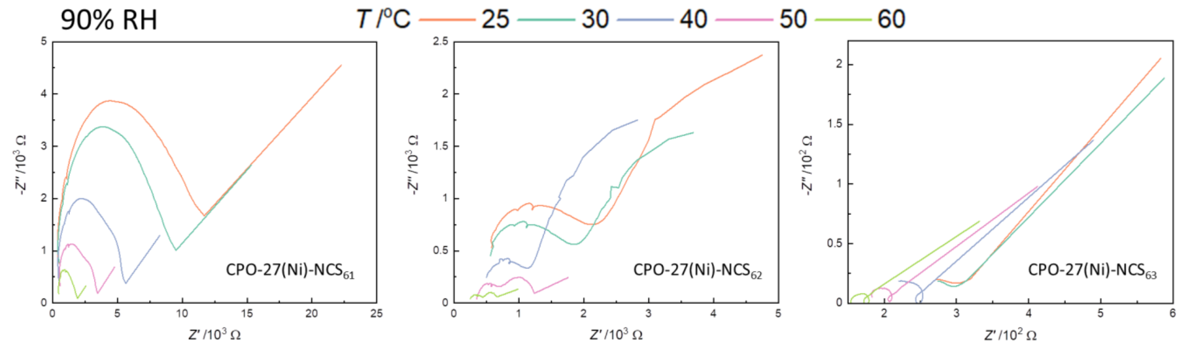

Figure S20. Variable temperature EIS conductivity measurements at 90% RH for **CPO-27(Ni)-NCS** with various stoichiometric ratios: 6:1, 6:2, 6:3 (**CPO-27** to NH_4SCN).

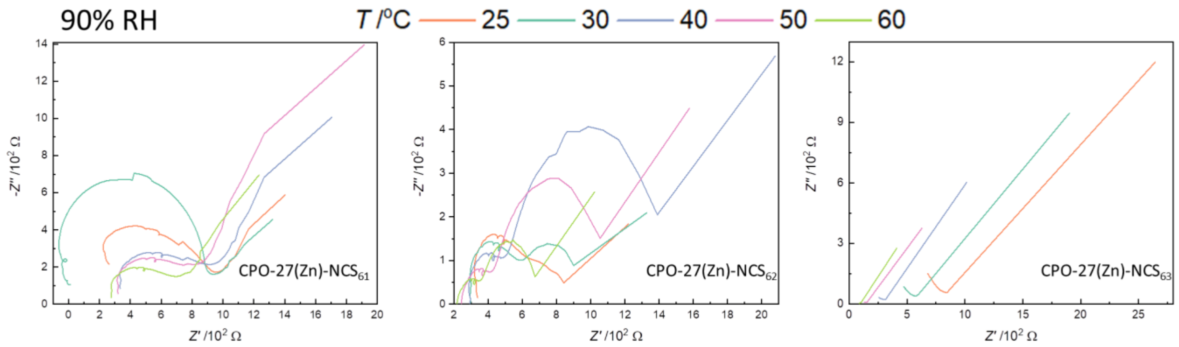

Figure S21. Variable temperature EIS conductivity measurements at 90% RH for **CPO-27(Zn)-NCS** with various stoichiometric ratios: 6:1, 6:2, 6:3 (**CPO-27** to NH_4SCN).

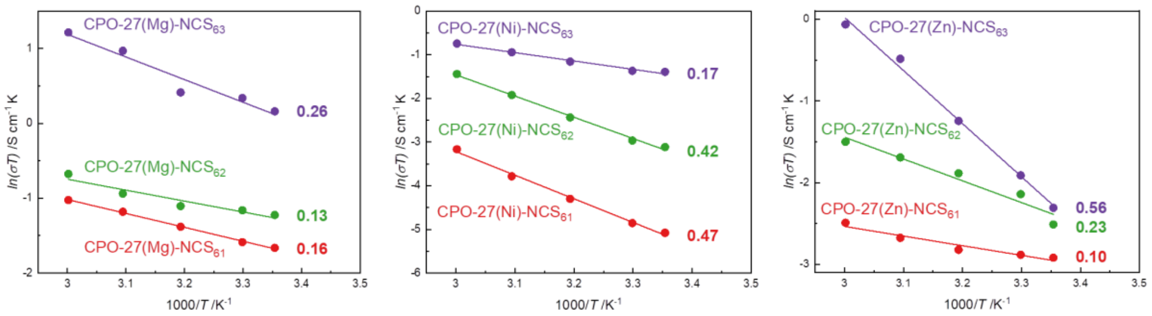

Figure S22. Arrhenius plots with activation energies indicated as numbers (in eV) at 90% RH for **CPO-27-NCS** with various stoichiometric amounts of NH_4SCN added; given as 6:1, 6:2, 6:3 **CPO-27** to NH_4SCN ratios.

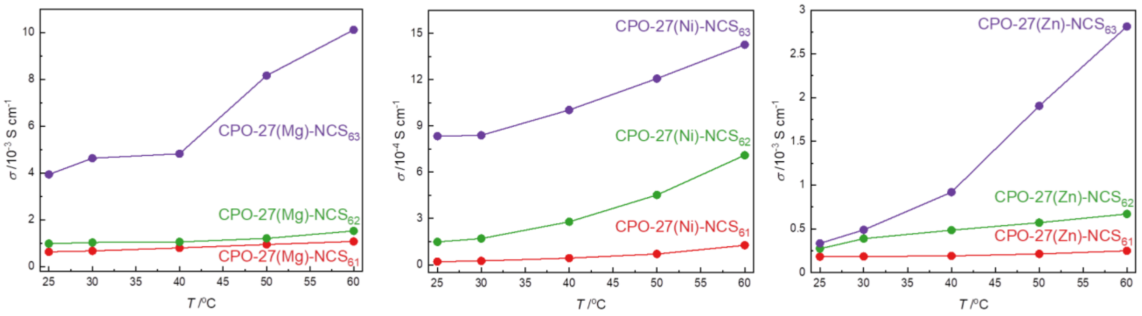

Figure S23. Proton conductivity change with temperature at 90% RH for **CPO-27-NCS** with various stoichiometric amounts of NH_4SCN added; given as 6:1, 6:2, 6:3 **CPO-27** to NH_4SCN ratios.

Table S2. Comparison of proton conductivities for **CPO-27-NCS**.

RH (%)	Sample	Conductivity ($S\cdot cm^{-1}$)					Activation energy (eV)
		25°C	30°C	40°C	50°C	60°C	
30	CPO-27(Zn)-NCS	$3,3\cdot 10^{-6}$	$8,0\cdot 10^{-6}$	$2,9\cdot 10^{-5}$	$8,2\cdot 10^{-5}$	$9,0\cdot 10^{-5}$	0,86
	CPO-27(Ni)-NCS	$2,2\cdot 10^{-6}$	$2,4\cdot 10^{-6}$	$2,6\cdot 10^{-6}$	$2,7\cdot 10^{-6}$	-	0,08
	CPO-27(Mg)-NCS	$7,0\cdot 10^{-5}$	$8,7\cdot 10^{-5}$	$1,4\cdot 10^{-4}$	$1,6\cdot 10^{-4}$	-	0,30
45	CPO-27(Zn)-NCS	$7,3\cdot 10^{-6}$	$1,2\cdot 10^{-5}$	$3,3\cdot 10^{-5}$	$1,2\cdot 10^{-4}$	$1,4\cdot 10^{-4}$	0,81
	CPO-27(Ni)-NCS	$3,3\cdot 10^{-6}$	$4,1\cdot 10^{-6}$	$5,2\cdot 10^{-6}$	$5,6\cdot 10^{-6}$	$6,7\cdot 10^{-6}$	0,19
	CPO-27(Mg)-NCS	$2,6\cdot 10^{-4}$	$3,3\cdot 10^{-4}$	$4,5\cdot 10^{-4}$	$5,3\cdot 10^{-4}$	$6,2\cdot 10^{-4}$	0,23
60	CPO-27(Zn)-NCS	$4,2\cdot 10^{-5}$	$7,1\cdot 10^{-5}$	$1,7\cdot 10^{-4}$	$3,7\cdot 10^{-4}$	$5,3\cdot 10^{-4}$	0,66
	CPO-27(Ni)-NCS	$1,0\cdot 10^{-5}$	$1,2\cdot 10^{-5}$	$2,0\cdot 10^{-5}$	$2,4\cdot 10^{-5}$	$2,5\cdot 10^{-5}$	0,27
	CPO-27(Mg)-NCS	$6,8\cdot 10^{-4}$	$7,4\cdot 10^{-4}$	$8,7\cdot 10^{-4}$	$1,1\cdot 10^{-3}$	$1,2\cdot 10^{-3}$	0,17
75	CPO-27(Zn)-NCS	$1,9\cdot 10^{-4}$	$2,8\cdot 10^{-4}$	$4,9\cdot 10^{-4}$	$8,1\cdot 10^{-4}$	$1,1\cdot 10^{-3}$	0,46
	CPO-27(Ni)-NCS	$8,5\cdot 10^{-5}$	$1,1\cdot 10^{-4}$	$2,1\cdot 10^{-4}$	$3,1\cdot 10^{-4}$	$3,7\cdot 10^{-4}$	0,40
	CPO-27(Mg)-NCS	$3,1\cdot 10^{-3}$	$3,3\cdot 10^{-3}$	$3,8\cdot 10^{-3}$	$4,5\cdot 10^{-3}$	$5,1\cdot 10^{-3}$	0,15
90	CPO-27(Zn)-NCS	$3,3\cdot 10^{-4}$	$4,9\cdot 10^{-4}$	$9,2\cdot 10^{-4}$	$1,9\cdot 10^{-3}$	$2,8\cdot 10^{-3}$	0,56
	CPO-27(Ni)-NCS	$8,3\cdot 10^{-4}$	$8,4\cdot 10^{-4}$	$1,0\cdot 10^{-3}$	$1,2\cdot 10^{-3}$	$1,4\cdot 10^{-3}$	0,17
	CPO-27(Mg)-NCS	$3,9\cdot 10^{-3}$	$4,6\cdot 10^{-3}$	$4,8\cdot 10^{-3}$	$8,2\cdot 10^{-3}$	$1,0\cdot 10^{-2}$	0,26

Table S3. Comparison of proton conductivities for **CPO-27(Mg)-NCS** with various with various stoichiometric amounts of NH_4SCN added; given as 6:1, 6:2, 6:3 **CPO-27** to NH_4SCN ratios.

RH (%)	Sample	Conductivity ($S\cdot cm^{-1}$)					Activation energy (eV)
		25°C	30°C	40°C	50°C	60°C	
90	CPO-27(Mg)-NCS_{61}	$6,4\cdot 10^{-4}$	$6,8\cdot 10^{-4}$	$8,0\cdot 10^{-4}$	$9,5\cdot 10^{-4}$	$1,1\cdot 10^{-3}$	0,16
	CPO-27(Mg)-NCS_{62}	$9,9\cdot 10^{-4}$	$1,0\cdot 10^{-3}$	$1,1\cdot 10^{-3}$	$1,2\cdot 10^{-3}$	$1,5\cdot 10^{-3}$	0,13
	CPO-27(Mg)-NCS_{63}	$3,9\cdot 10^{-3}$	$4,6\cdot 10^{-3}$	$4,8\cdot 10^{-3}$	$8,2\cdot 10^{-3}$	$1,0\cdot 10^{-2}$	0,26

Table S4. Comparison of proton conductivities for **CPO-27(Ni)-NCS** with various with various stoichiometric amounts of NH_4SCN added; given as 6:1, 6:2, 6:3 **CPO-27** to NH_4SCN ratios.

RH (%)	Sample	Conductivity ($S\cdot cm^{-1}$)					Activation energy (eV)
		25°C	30°C	40°C	50°C	60°C	
90	CPO-27(Ni)-NCS_{61}	$2,1\cdot 10^{-5}$	$2,6\cdot 10^{-5}$	$4,3\cdot 10^{-5}$	$7,0\cdot 10^{-5}$	$1,3\cdot 10^{-4}$	0,47
	CPO-27(Ni)-NCS_{62}	$1,5\cdot 10^{-4}$	$1,7\cdot 10^{-4}$	$2,8\cdot 10^{-4}$	$4,5\cdot 10^{-4}$	$7,1\cdot 10^{-4}$	0,42
	CPO-27(Ni)-NCS_{63}	$8,3\cdot 10^{-4}$	$8,4\cdot 10^{-4}$	$1,0\cdot 10^{-3}$	$1,2\cdot 10^{-3}$	$1,4\cdot 10^{-3}$	0,17

Table S5. Comparison of proton conductivities for **CPO-27(Zn)-NCS** with various with various stoichiometric amounts of NH_4SCN added; given as 6:1, 6:2, 6:3 **CPO-27** to NH_4SCN ratios.

RH (%)	Sample	Conductivity ($S\cdot cm^{-1}$)					Activation energy (eV)
		25°C	30°C	40°C	50°C	60°C	
90	CPO-27(Zn)-NCS_{61}	$1,8\cdot 10^{-4}$	$1,9\cdot 10^{-4}$	$1,9\cdot 10^{-4}$	$2,1\cdot 10^{-4}$	$2,5\cdot 10^{-4}$	0,10
	CPO-27(Zn)-NCS_{62}	$2,7\cdot 10^{-4}$	$3,9\cdot 10^{-4}$	$4,8\cdot 10^{-4}$	$5,7\cdot 10^{-4}$	$6,7\cdot 10^{-4}$	0,23
	CPO-27(Zn)-NCS_{63}	$3,3\cdot 10^{-4}$	$4,9\cdot 10^{-4}$	$9,2\cdot 10^{-4}$	$1,9\cdot 10^{-3}$	$2,8\cdot 10^{-3}$	0,56

Table S6. Proton conductivities for selected MOFs including those with protonated amines/ammonia and those based on **CPO-27**(MOF-74) materials.

Sample	Conductivity (S cm^{-1})	Conditions	Ref
$(Me_2NH_2)_3(SO_4)]_2[Zn_2(ox)_3]$	$4{,}2\cdot10^{-2}$	98% RH (25°C)	7
MOF-74(Mg)-Urea	$3{,}7\cdot10^{-2}$	95% RH (55°C)	8
VNU-15 $Fe_4(BDC)_2(NDC)(SO_4)_4(DMA)_4$	$2{,}9\cdot10^{-2}$	60% RH (95°C)	9
MROF-1 $[Me_2NH_2][In(thb)(pbdc)]\cdot2.5DMF\cdot3.5H_2O$	$1{,}7\cdot10^{-2}$	97% RH (70°C)	10
**CPO-27(Mg)-NCS**	$1{,}0\cdot10^{-2}$	90% RH (60°C)	this work
PCMOF-20 $(DMA)_3[Zr(HL)F_2]$ H_6L = 2,4,6-tris(4-phosphonophenyl) pyridine	$1{,}0\cdot10^{-2}$	95% RH (85°C)	11
$(Me_2NH_2)[Eu(L)]$	$3{,}8\cdot10^{-3}$	98% RH (100°C)	12
**CPO-27(Zn)-NCS**	$2{,}8\cdot10^{-3}$	90% RH (60°C)	this work
MOF-74(Ni)-Urea	$2{,}3\cdot10^{-3}$	95% RH (55°C)	8
**CPO-27(Ni)-NCS**	$1{,}4\cdot10^{-3}$	90% RH (60°C)	this work
$\{[Co_2Cl_2(BTC)_{4/3}]\cdot(Me_2NH_2)^{+2}\cdot4/3H_2O\}_n$	$1{,}2\cdot10^{-3}$	60% RH (50°C)	13
NH_4Br@HKUST-1	$9{,}0\cdot10^{-4}$	99% RH (25°C)	14
Ti-CAT-5 $Ti(THO)(DMA)_2$	$8{,}2\cdot10^{-4}$	98% RH (25°C)	15
$NMe_3(CH_2COOH)[FeCr(ox)_3]$	$8{,}0\cdot10^{-4}$	60% RH (25°C)	16
CPM-102 $[In(imdcH)\text{-}(ox)]\cdot(NH_4)^+(H_2O)_{1.5}$	$8{,}0\cdot10^{-4}$	98,5% RH (22,5°C)	17
$\{NH(prol)_3\}[MCr(ox)_3]$ ($M=Mn^{II}$, Fe^{II}, Co^{II}; $NH(prol)_3^+$=tri(3-hydroxypropyl) ammonium)	$1{,}0\cdot10^{-4}$	75% RH (25°C)	18
MOF-74(Ni)	$1{,}7\cdot10^{-5}$	95% RH (55°C)	8
MOF-74(Ni)-H_2O-Urea	$1{,}1\cdot10^{-5}$	95% RH (55°C)	8
$(NH_4)_3[Co_2(bamdpH)_2(HCOO)(H_2O)_2]$	$8{,}0\cdot10^{-6}$	90% RH (25°C)	19
MOF-74(Co)	$4{,}5\cdot10^{-3}$ *	95% RH (90°C)	20
MOF-74(Co)	$1{,}4\cdot10^{-3}$ *	90% RH (60°C)	21

* proton conductivity along the [001] channels, measured on single crystals

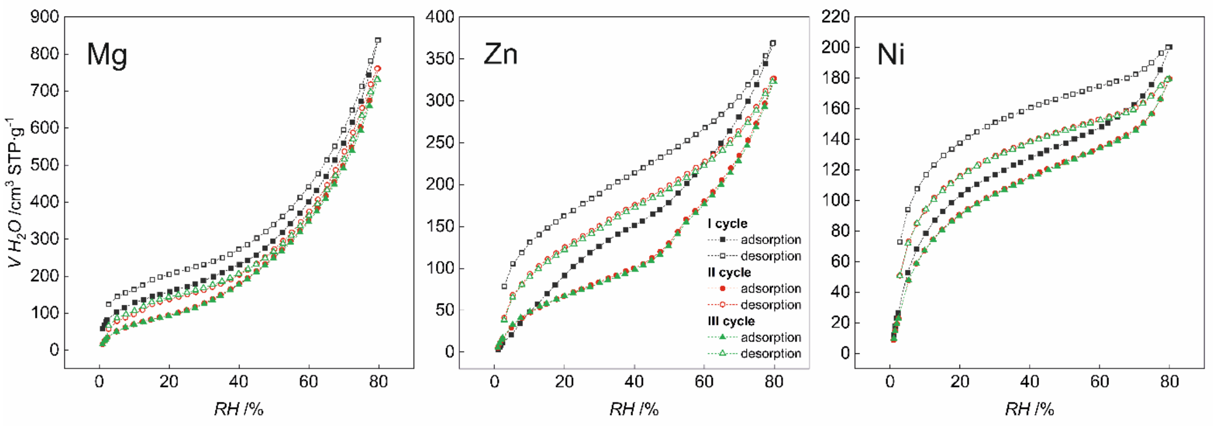

Figure S24. H_2O adsorption cycling test for **CPO-27-NCS**.

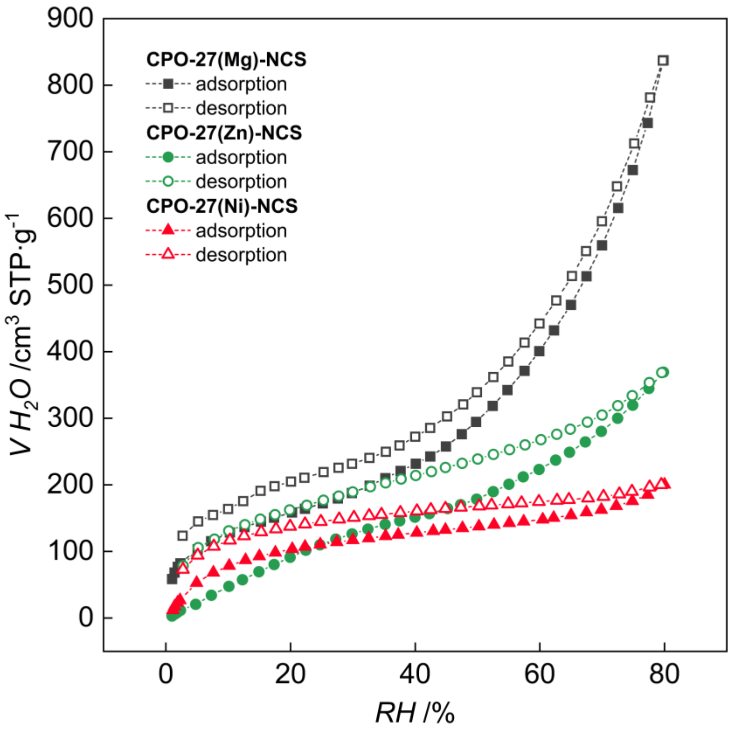

Figure S25. H_2O vapor adsorption measurement at 298 K for **CPO-27-NCS**, closed symbols: adsorption, open symbols desorption.

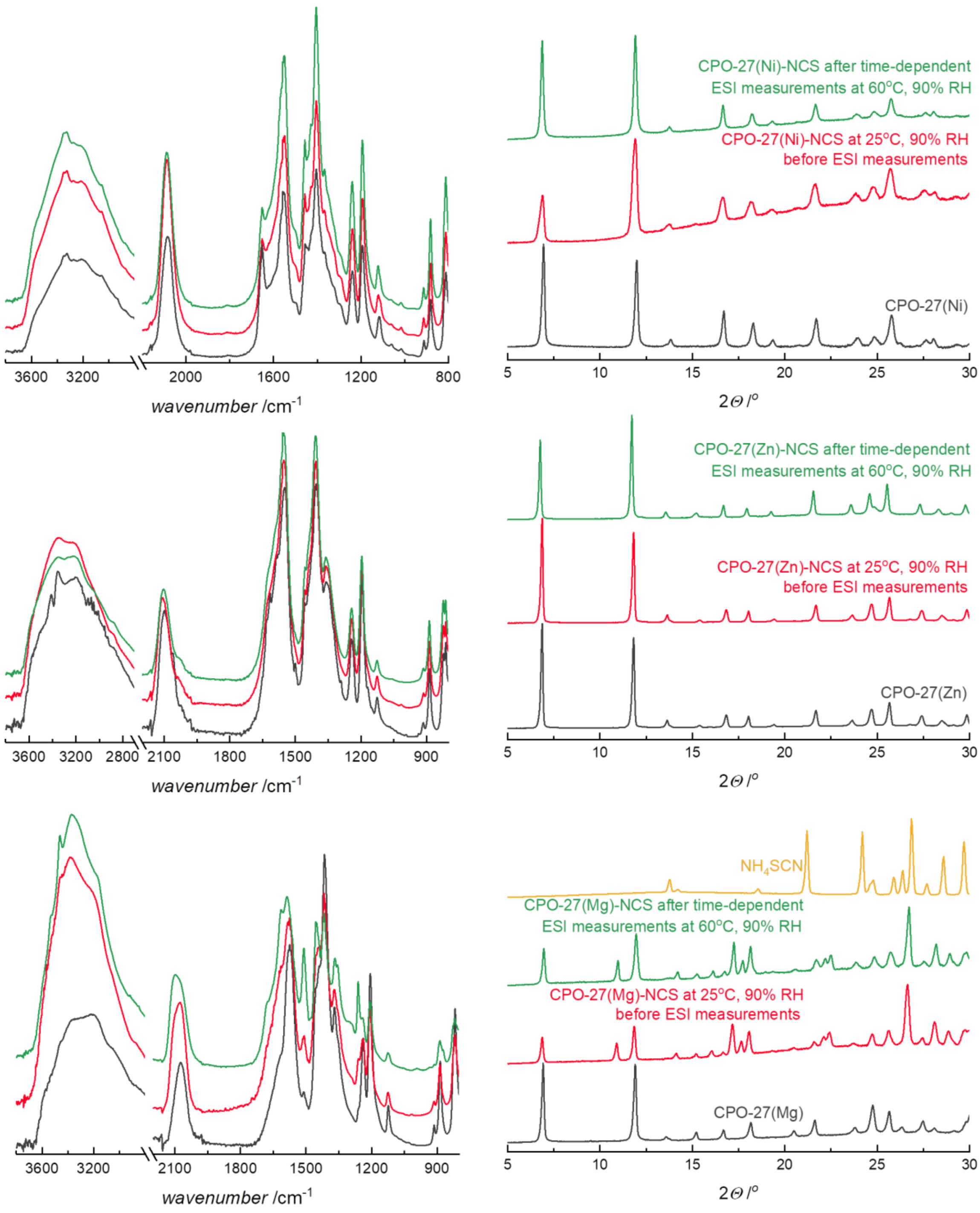

Figure S26. IR spectra (left) and PXRD patterns (right) of the as-synthesized **CPO-27-NCS** compared with those of samples conditioned at 90% RH before EIS measurement (red) and after EIS measurement (green).

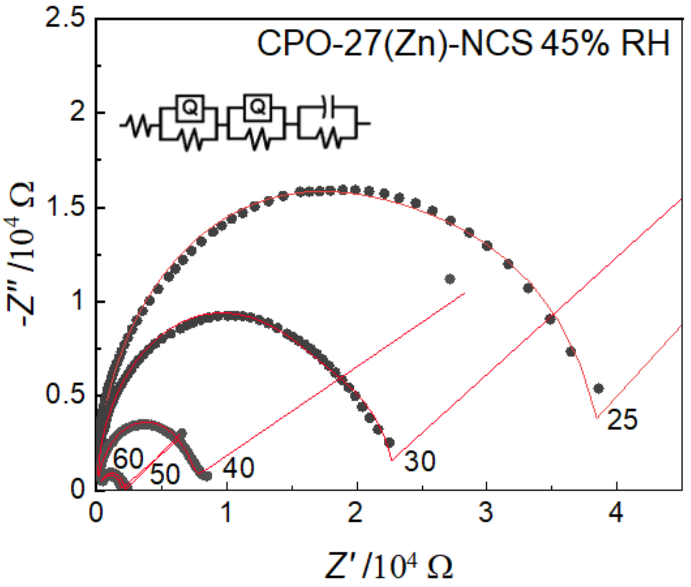

Figure S27. Representative temperature-dependent AC impedance plots at 45% RH for **CPO-27(Zn)-NCS** (numbers indicate temperatures in ºC) with the equivalent circuit used for fitting the plots (ZSimpWin software).

The equivalent circuit proposal indicates the presence of components which may refer to bulk resistance, different orientation of crystal planes contribution, grain boundary resistance and electrode/electrolyte interface.

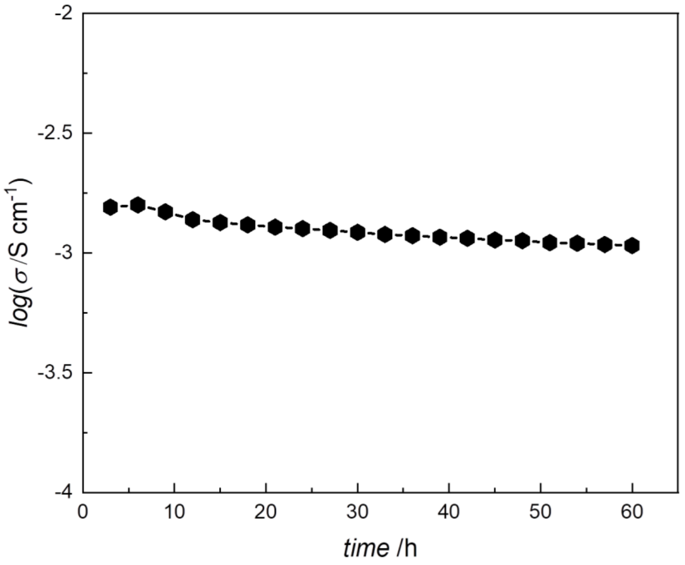

Figure S28. Time-dependent proton conductivity of **CPO-27(Mg)-NCS** at 60°C and 90% RH.

References in the Supporting Information

(1) Choi, I.; Jung, Y. E.; Yoo, S. J.; Kim, J. Y.; Kim, H.-J.; Lee, C. Y.; Jang, J. H. Facile Synthesis of M-MOF-74 (M=Co, Ni, Zn) and Its Application as an ElectroCatalyst for Electrochemical CO2 Conversion and H2 Production. *J. Electrochem. Sci. Technol.* **2017**, *8* (1), 61–68. https://doi.org/10.33961/JECST.2017.8.1.61.

(2) Caskey, S. R.; Wong-Foy, A. G.; Matzger, A. J. Dramatic Tuning of Carbon Dioxide Uptake via Metal Substitution in a Coordination Polymer with Cylindrical Pores. *J. Am. Chem. Soc.* **2008**, *130* (33), 10870–10871. https://doi.org/10.1021/ja8036096.
(3) Delley, B. From Molecules to Solids with the DMol3 Approach. *J. Chem. Phys.* **2000**, *113* (18), 7756–7764. https://doi.org/10.1063/1.1316015.
(4) Perdew, J. P.; Burke, K.; Ernzerhof, M. Generalized Gradient Approximation Made Simple. *Phys. Rev. Lett.* **1996**, *77* (18), 3865–3868. https://doi.org/10.1103/PhysRevLett.77.3865.
(5) Hammer, B.; Hansen, L. B.; Nørskov, J. K. Improved Adsorption Energetics within Density-Functional Theory Using Revised Perdew-Burke-Ernzerhof Functionals. *Phys. Rev. B* **1999**, *59* (11), 7413–7421. https://doi.org/10.1103/PhysRevB.59.7413.
(6) Pato-Doldán, B.; Rosnes, M. H.; Dietzel, P. D. C. An In-Depth Structural Study of the Carbon Dioxide Adsorption Process in the Porous Metal-Organic Frameworks CPO-27-M. *ChemSusChem* **2017**, *10* (8), 1710–1719. https://doi.org/10.1002/cssc.201601752.
(7) Nagarkar, S. S.; Unni, S. M.; Sharma, A.; Kurungot, S.; Ghosh, S. K. Two-in-One: Inherent Anhydrous and Water-Assisted High Proton Conduction in a 3D Metal-Organic Framework. *Angew. Chem. Int. Ed.* **2014**, *53* (10), 2638–2642. https://doi.org/10.1002/anie.201309077.
(8) Sarango-Ramírez, M. K.; Lim, D.-W.; Kolokolov, D. I.; Khudozhitkov, A. E.; Stepanov, A. G.; Kitagawa, H. Superprotonic Conductivity in Metal–Organic Framework via Solvent-Free Coordinative Urea Insertion. *J. Am. Chem. Soc.* **2020**, *142* (15), 6861–6865. https://doi.org/10.1021/jacs.0c00303.
(9) Tu, T. N.; Phan, N. Q.; Vu, T. T.; Nguyen, H. L.; Cordova, K. E.; Furukawa, H. High Proton Conductivity at Low Relative Humidity in an Anionic Fe-Based Metal–Organic Framework. *J. Mater. Chem. A* **2016**, *4* (10), 3638–3641. https://doi.org/10.1039/C5TA10467J.
(10) Han, Y.-H.; Ye, Y.; Tian, C.; Zhang, Z.; Du, S.-W.; Xiang, S. High Proton Conductivity in an Unprecedented Anionic Metalloring Organic Framework (MROF) Containing Novel Metalloring Clusters with the Largest Diameter. *J. Mater. Chem. A* **2016**, *4* (48), 18742–18746. https://doi.org/10.1039/C6TA07939C.
(11) Hassanzadeh Fard, Z.; Wong, N. E.; Malliakas, C. D.; Ramaswamy, P.; Taylor, J. M.; Otsubo, K.; Shimizu, G. K. H. Superprotonic Phase Change to a Robust Phosphonate Metal–Organic Framework. *Chem. Mater.* **2018**, *30* (2), 314–318. https://doi.org/10.1021/acs.chemmater.7b04467.
(12) Wei, Y.-S.; Hu, X.-P.; Han, Z.; Dong, X.-Y.; Zang, S.-Q.; Mak, T. C. W. Unique Proton Dynamics in an Efficient MOF-Based Proton Conductor. *J. Am. Chem. Soc.* **2017**, *139* (9), 3505–3512. https://doi.org/10.1021/jacs.6b12847.
(13) Liu, S.-J.; Cao, C.; Yang, F.; Yu, M.-H.; Yao, S.-L.; Zheng, T.-F.; He, W.-W.; Zhao, H.-X.; Hu, T.-L.; Bu, X.-H. High Proton Conduction in Two Co ^{II} and Mn ^{II} Anionic Metal–Organic Frameworks Derived from 1,3,5-Benzenetricarboxylic Acid. *Cryst. Growth Des.* **2016**, *16* (12), 6776–6780. https://doi.org/10.1021/acs.cgd.6b00776.
(14) You, Y.-W.; Xue, C.; Tian, Z.-F.; Liu, S.-X.; Ren, X.-M. Three Orders of Magnitude Enhancement of Proton Conductivity of Porous Coordination Polymers by Incorporating Ion-Pairs into a Framework. *Dalton Trans.* **2016**, *45* (18), 7893–7899. https://doi.org/10.1039/C6DT00290K.
(15) Nguyen, N. T. T.; Furukawa, H.; Gándara, F.; Trickett, C. A.; Jeong, H. M.; Cordova, K. E.; Yaghi, O. M. Three-Dimensional Metal-Catecholate Frameworks and Their Ultrahigh Proton Conductivity. *J. Am. Chem. Soc.* **2015**, *137* (49), 15394–15397. https://doi.org/10.1021/jacs.5b10999.
(16) Sadakiyo, M.; Ōkawa, H.; Shigematsu, A.; Ohba, M.; Yamada, T.; Kitagawa, H. Promotion of Low-Humidity Proton Conduction by Controlling Hydrophilicity in Layered Metal–Organic Frameworks. *J. Am. Chem. Soc.* **2012**, *134* (12), 5472–5475. https://doi.org/10.1021/ja300122r.
(17) Zhao, X.; Mao, C.; Bu, X.; Feng, P. Direct Observation of Two Types of Proton Conduction Tunnels Coexisting in a New Porous Indium–Organic Framework. *Chem. Mater.* **2014**, *26* (8), 2492–2495. https://doi.org/10.1021/cm500473f.
(18) Ōkawa, H.; Shigematsu, A.; Sadakiyo, M.; Miyagawa, T.; Yoneda, K.; Ohba, M.; Kitagawa, H. Oxalate-Bridged Bimetallic Complexes {NH(Prol) $_3$ }[MCr(Ox) $_3$] (M = Mn ^{II} , Fe ^{II} , Co ^{II} ; NH(Prol) $_3$ $^+$ = Tri(3-Hydroxypropyl)Ammonium) Exhibiting Coexistent Ferromagnetism and

Proton Conduction. *J. Am. Chem. Soc.* **2009**, *131* (37), 13516–13522. https://doi.org/10.1021/ja905368d.

(19) Cai, Z.-S.; Bao, S.-S.; Wang, X.-Z.; Hu, Z.; Zheng, L.-M. Multiple-Step Humidity-Induced Single-Crystal to Single-Crystal Transformations of a Cobalt Phosphonate: Structural and Proton Conductivity Studies. *Inorg. Chem.* **2016**, *55* (7), 3706–3712. https://doi.org/10.1021/acs.inorgchem.6b00413.

(20) Hwang, S.; Lee, E. J.; Song, D.; Jeong, N. C. High Proton Mobility with High Directionality in Isolated Channels of MOF-74. *ACS Appl. Mater. Interfaces* **2018**, *10* (41), 35354–35360. https://doi.org/10.1021/acsami.8b11816.

(21) Javed, A.; Strauss, I.; Bunzen, H.; Caro, J.; Tiemann, M. Humidity-Mediated Anisotropic Proton Conductivity through the 1D Channels of Co-MOF-74. *Nanomaterials* **2020**, *10* (7), 1263. https://doi.org/10.3390/nano10071263.
